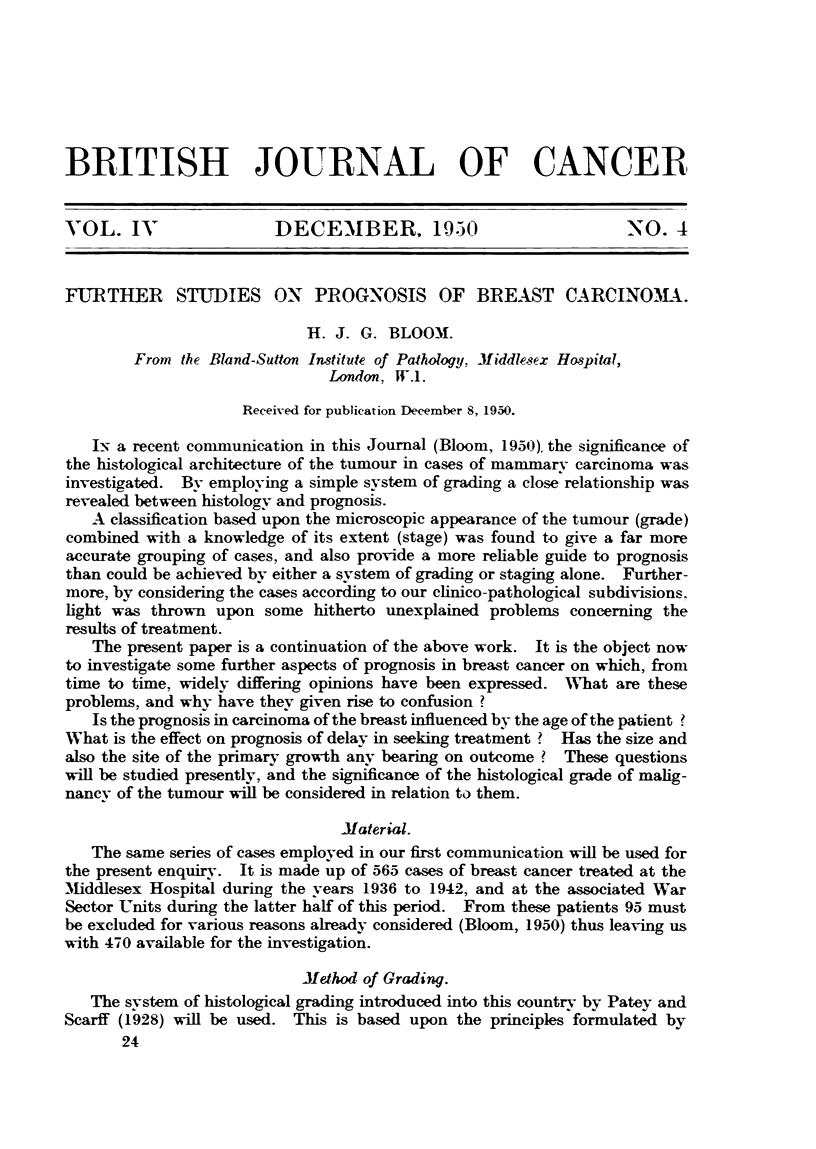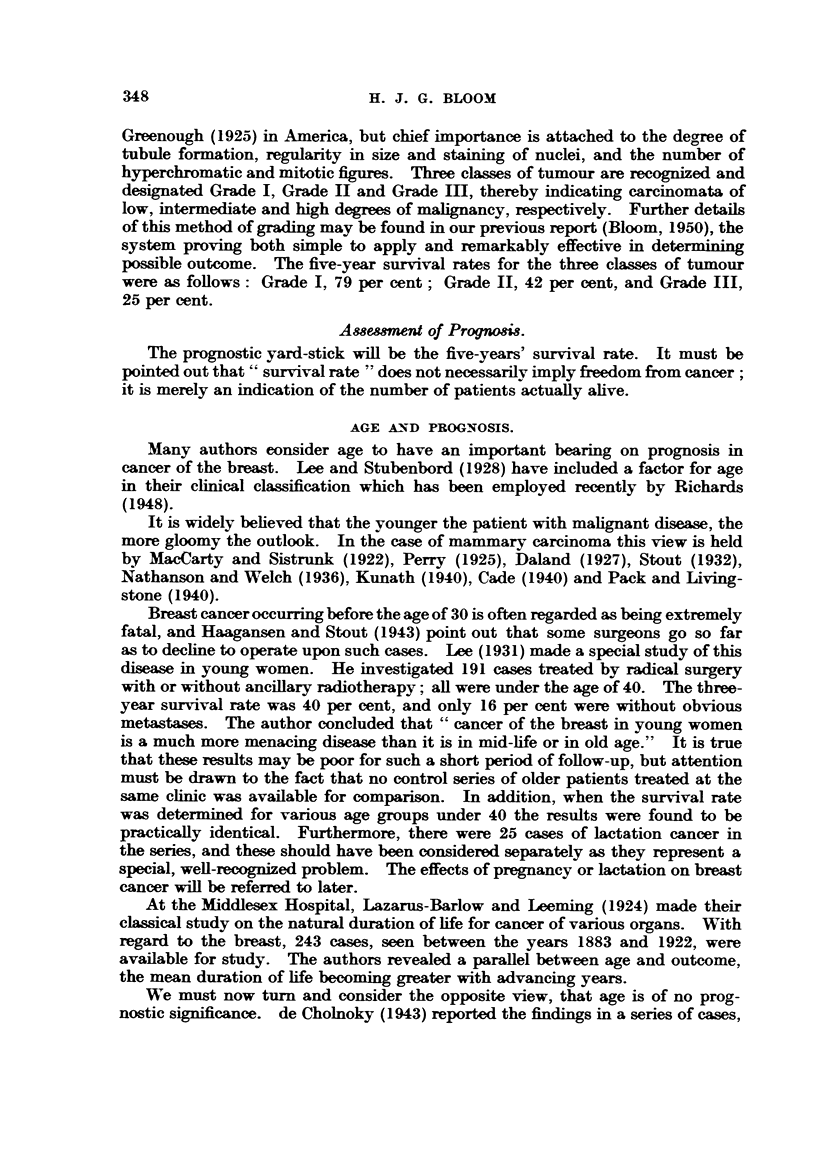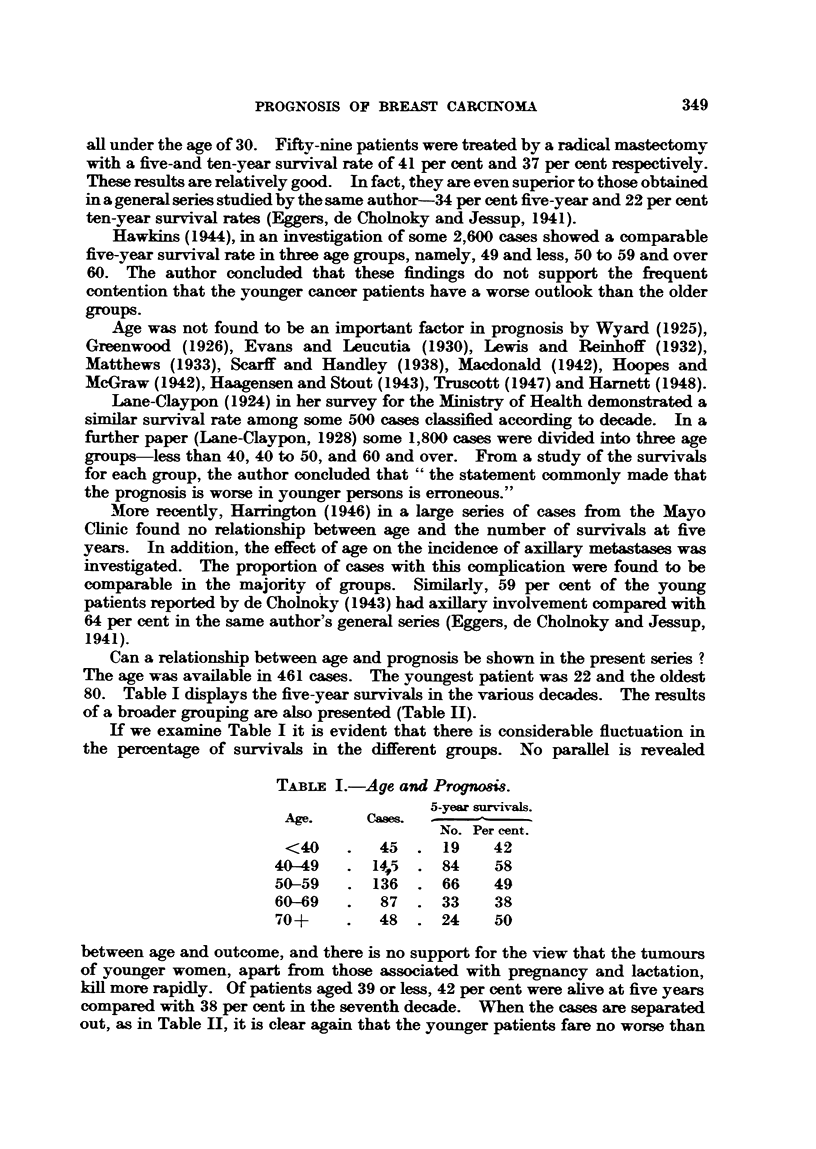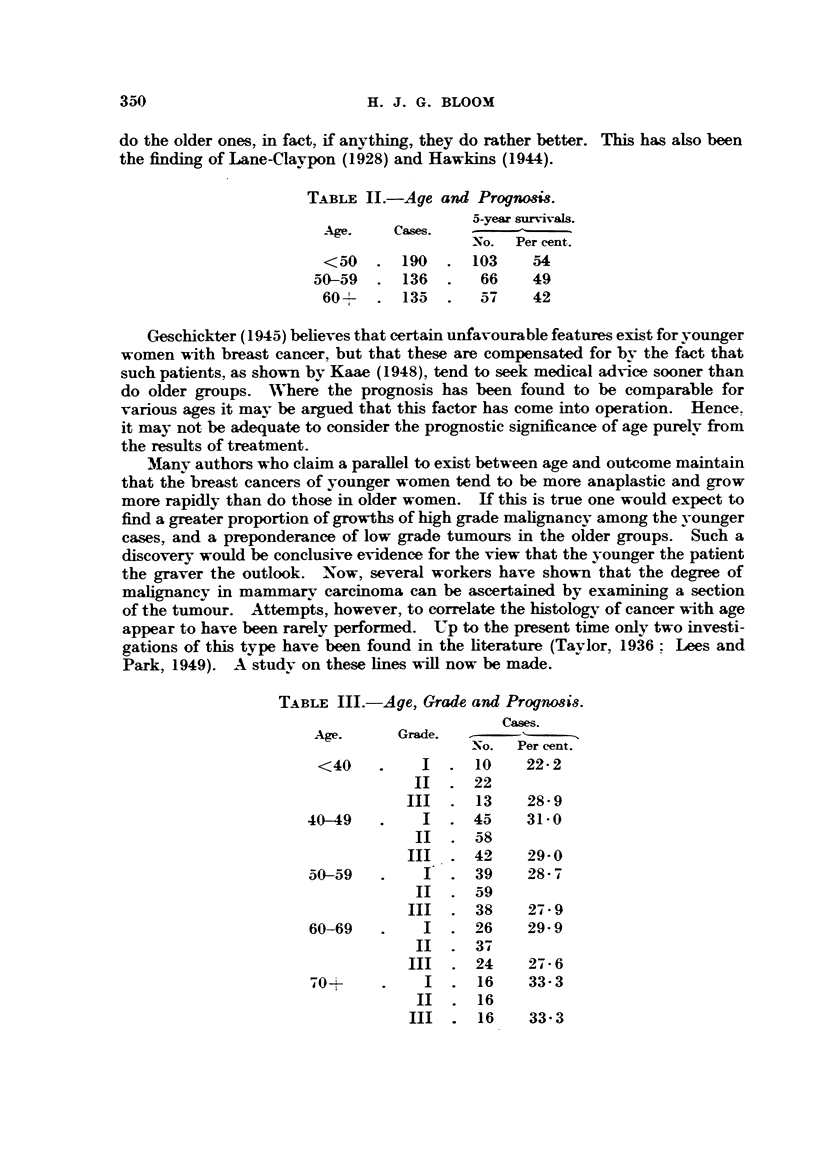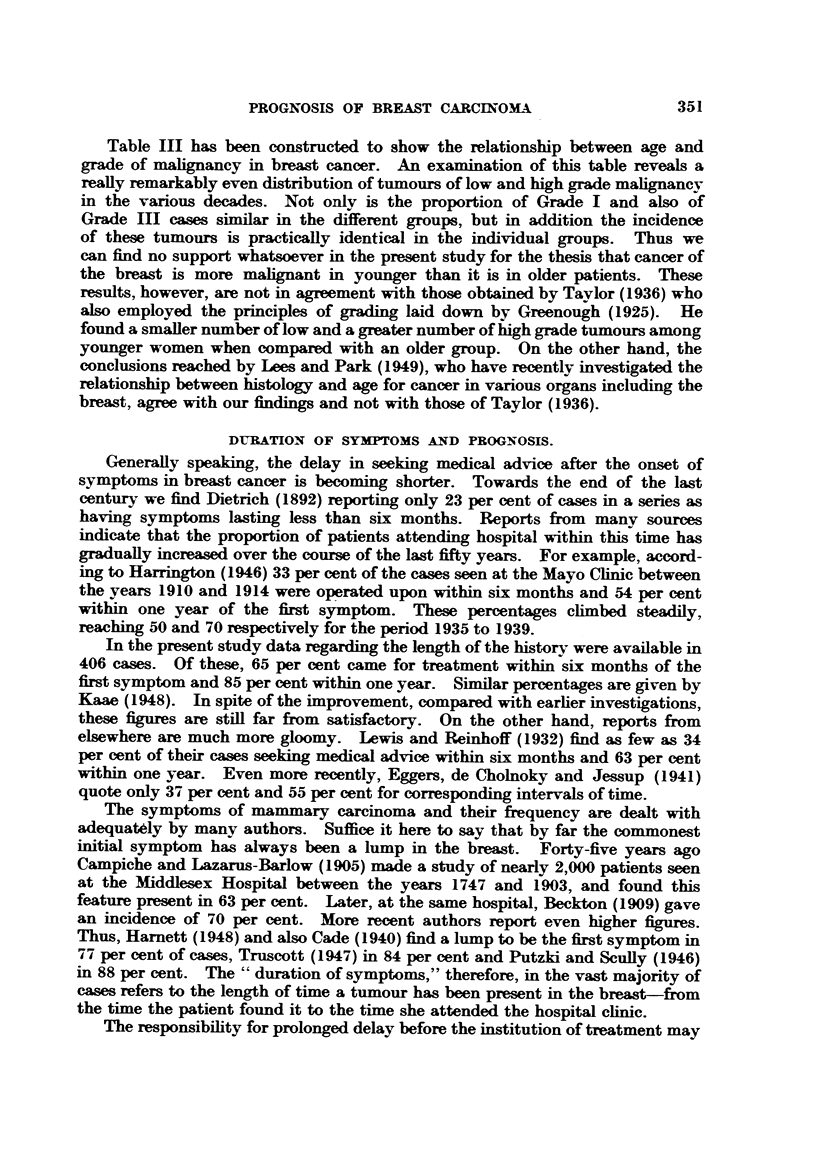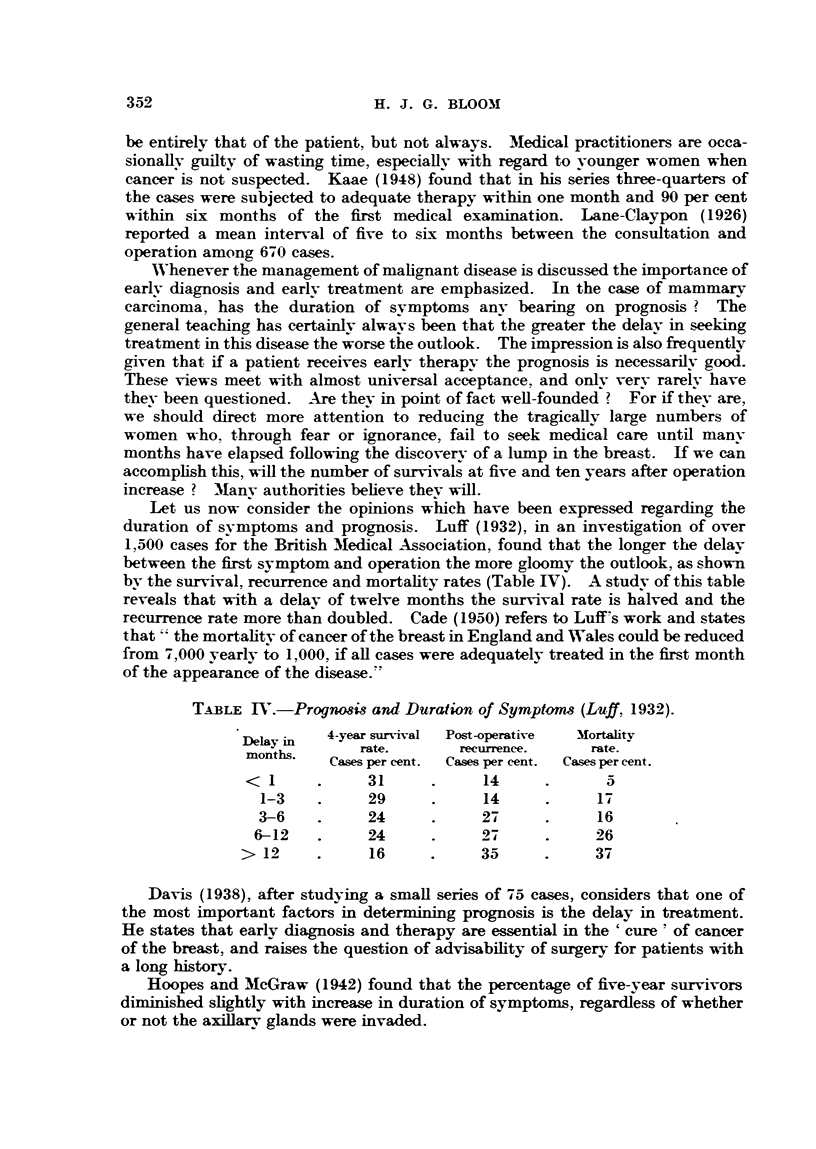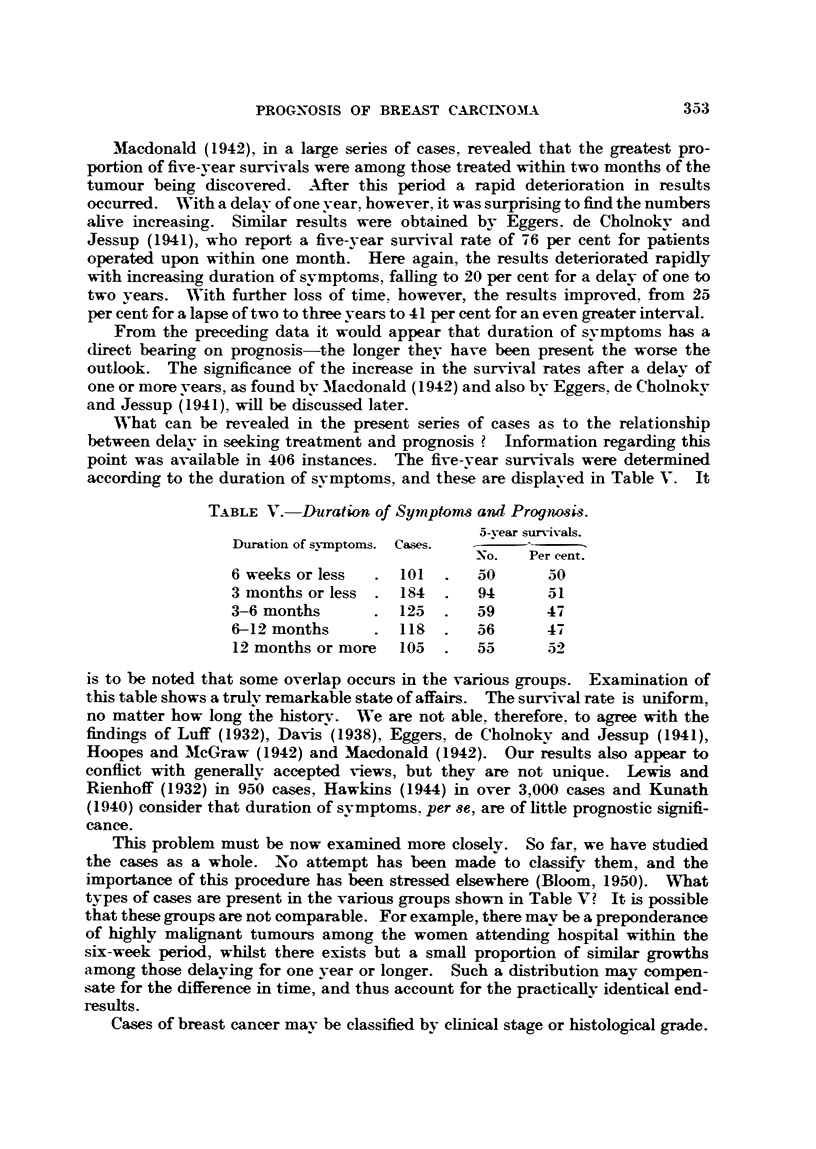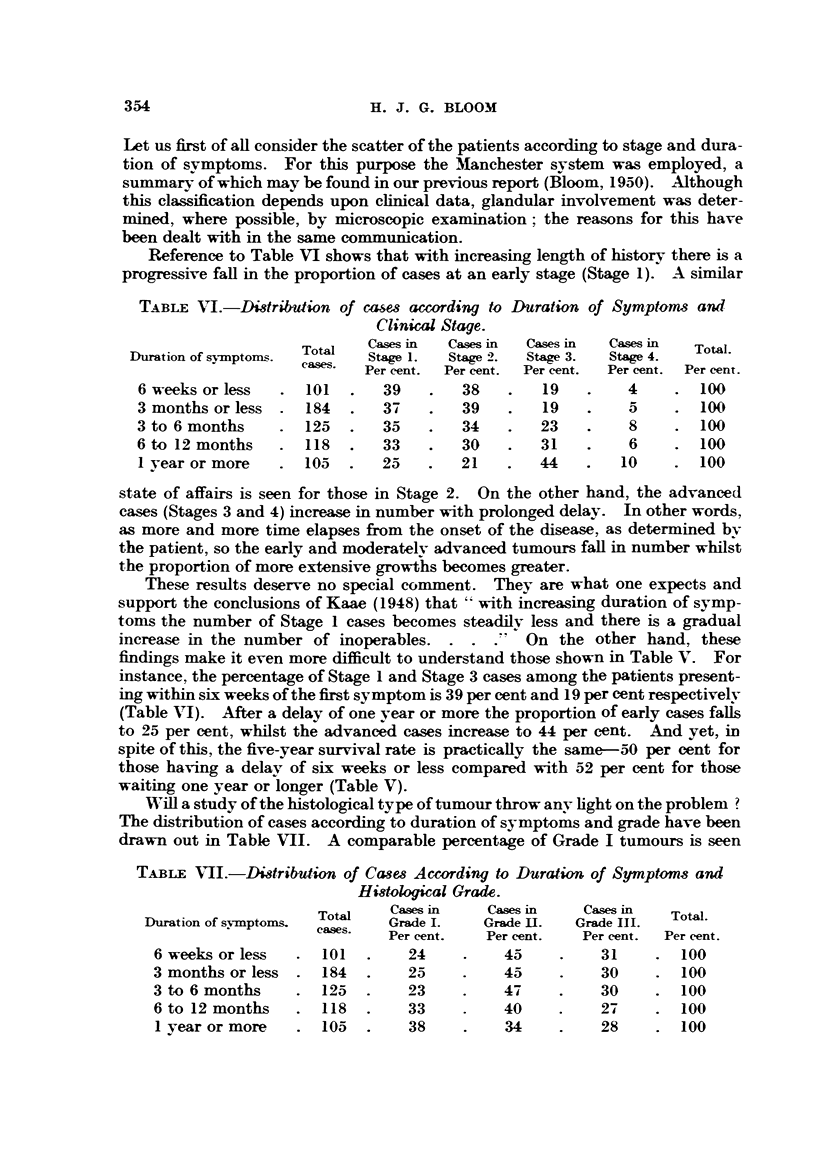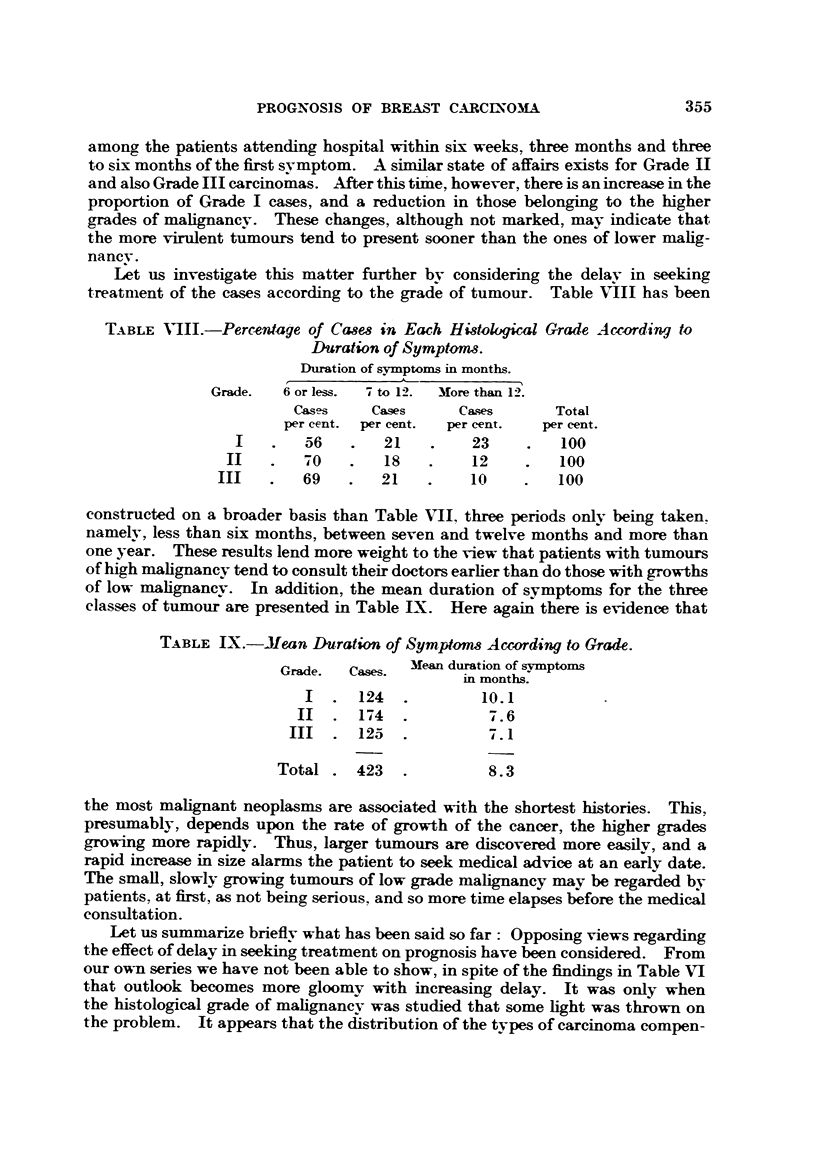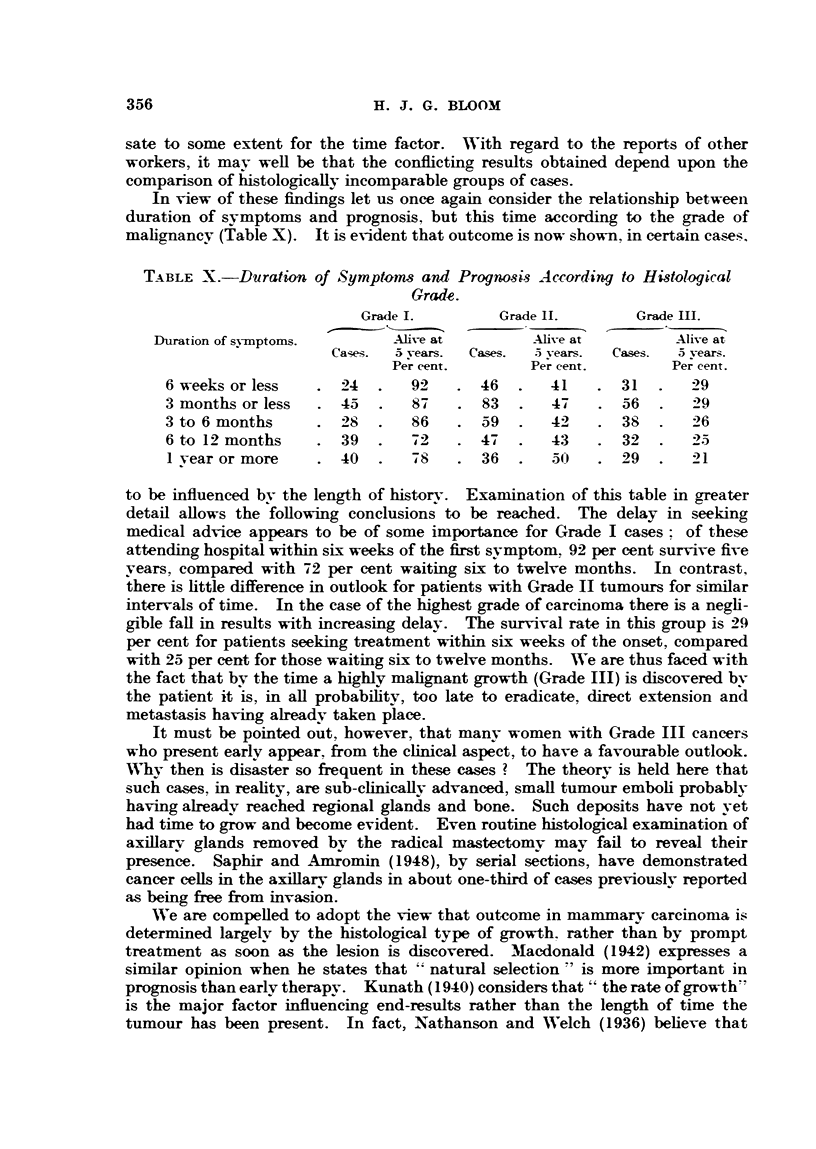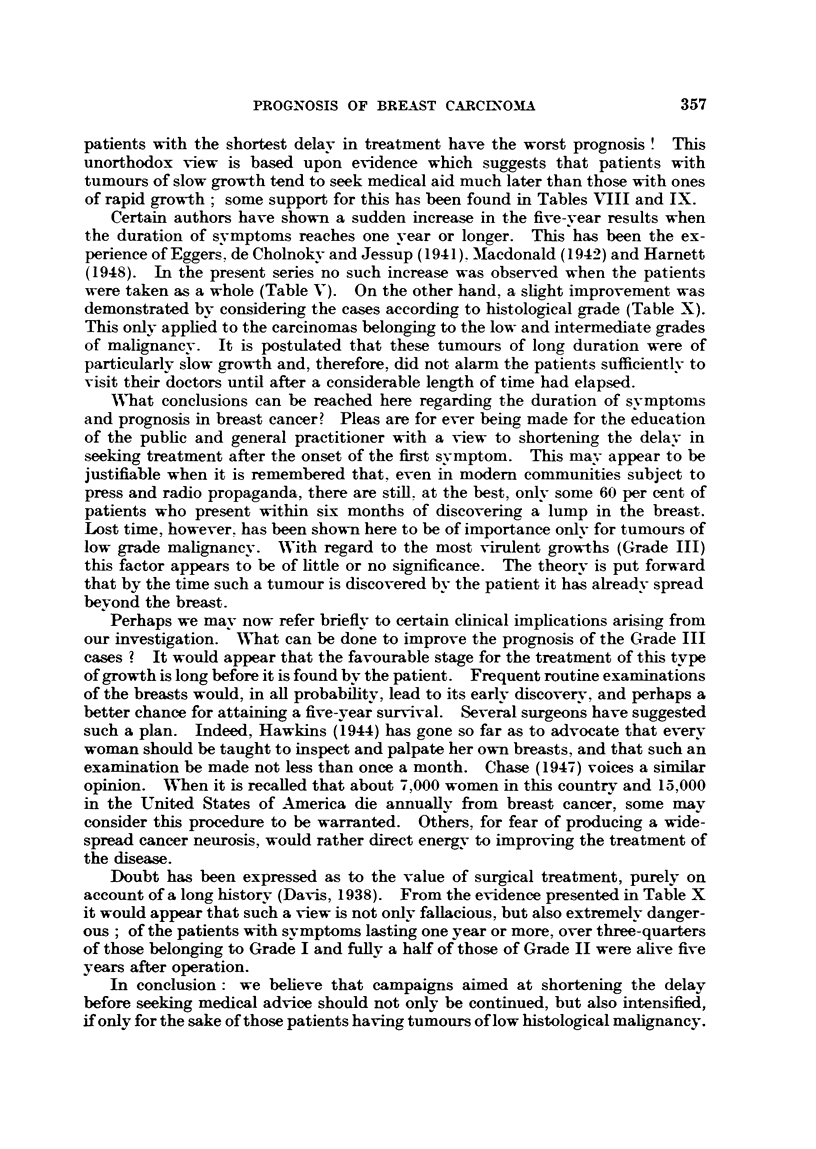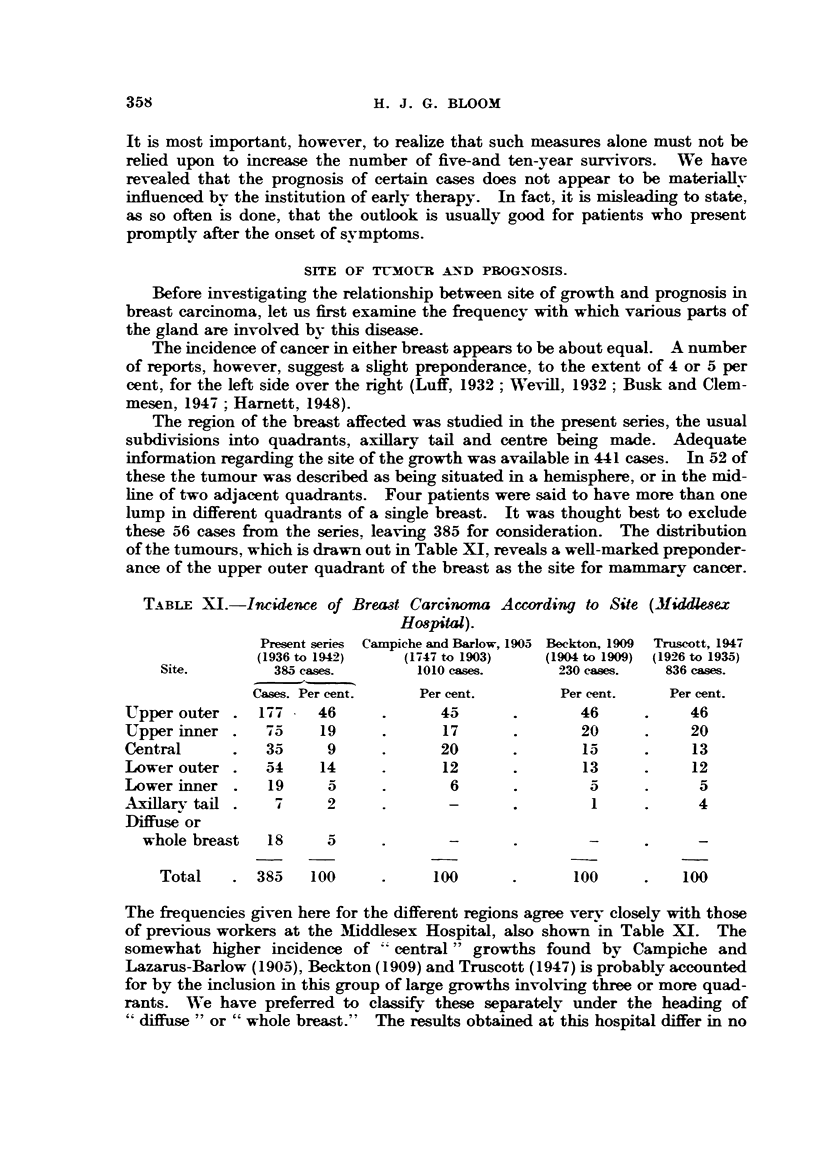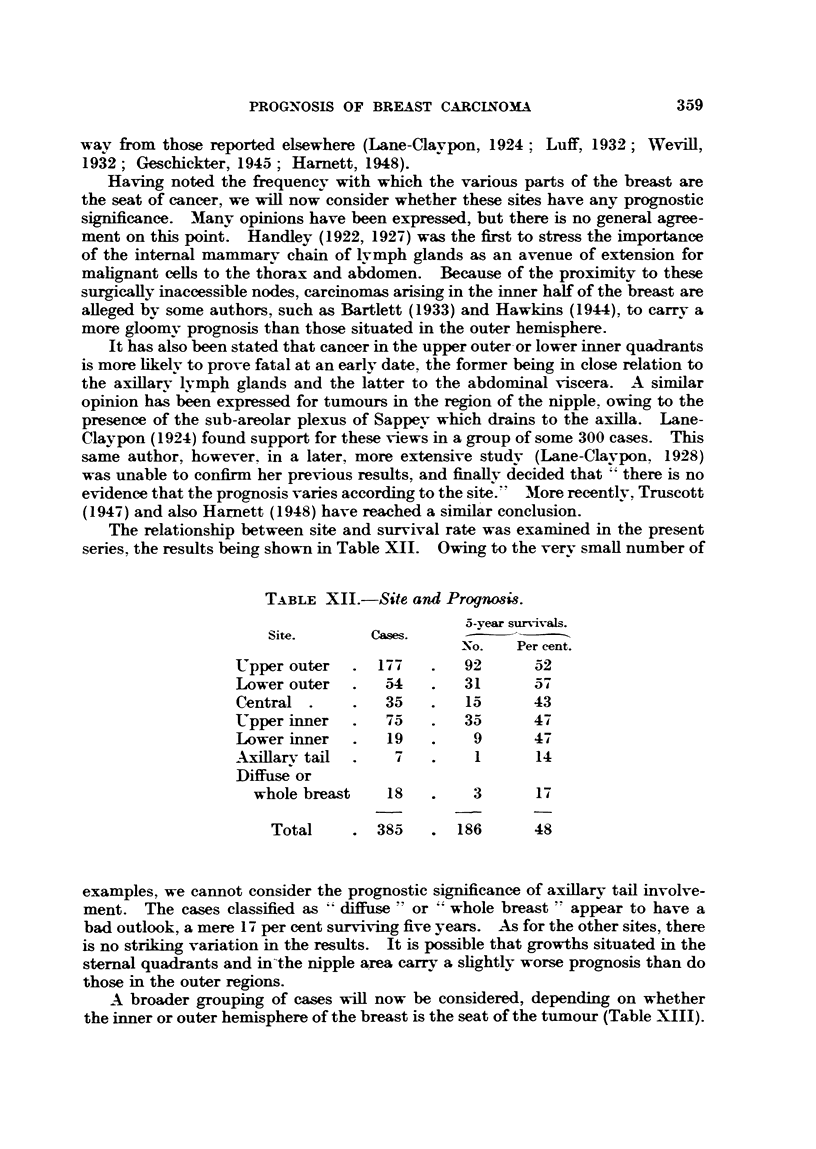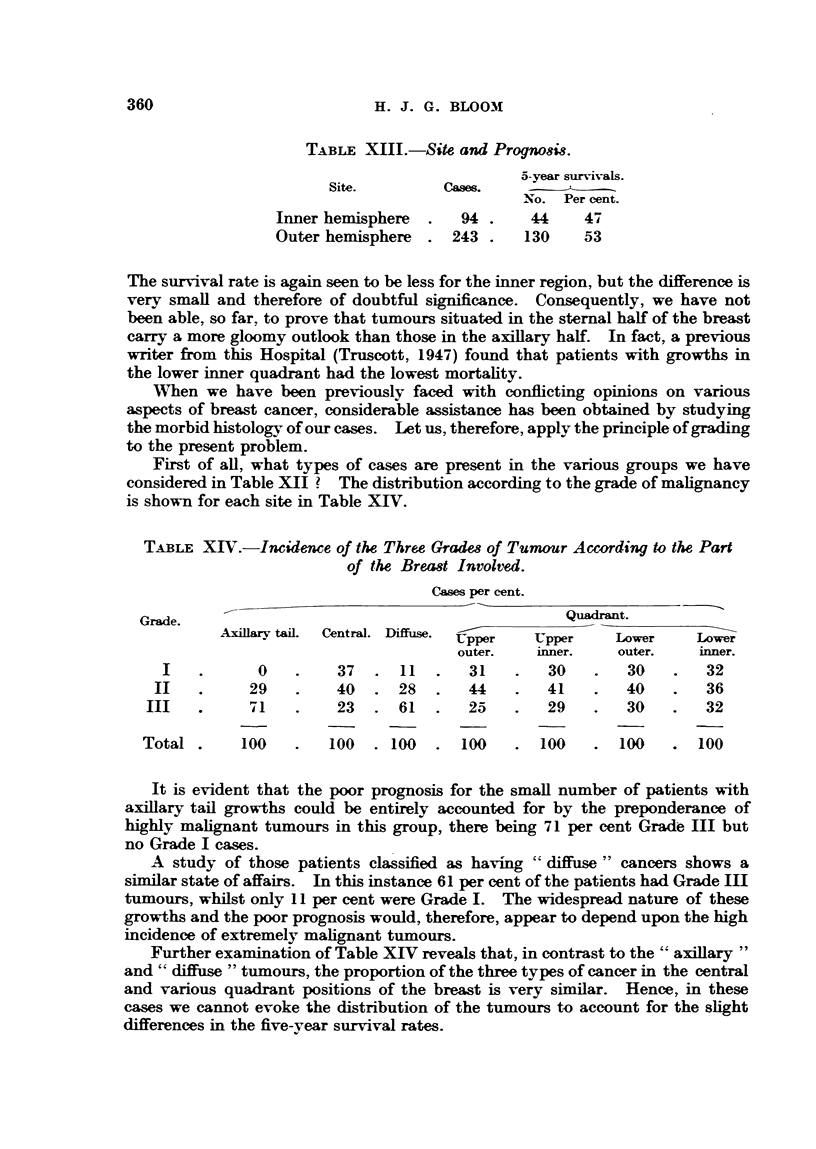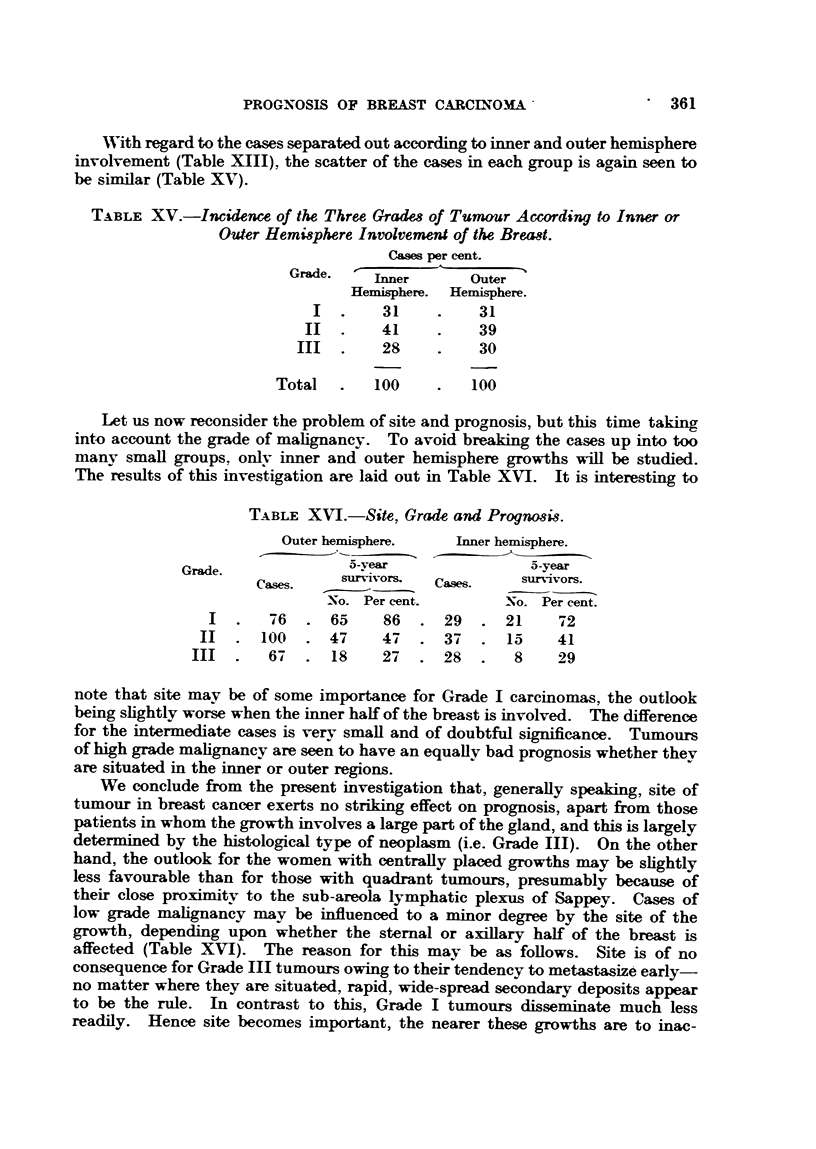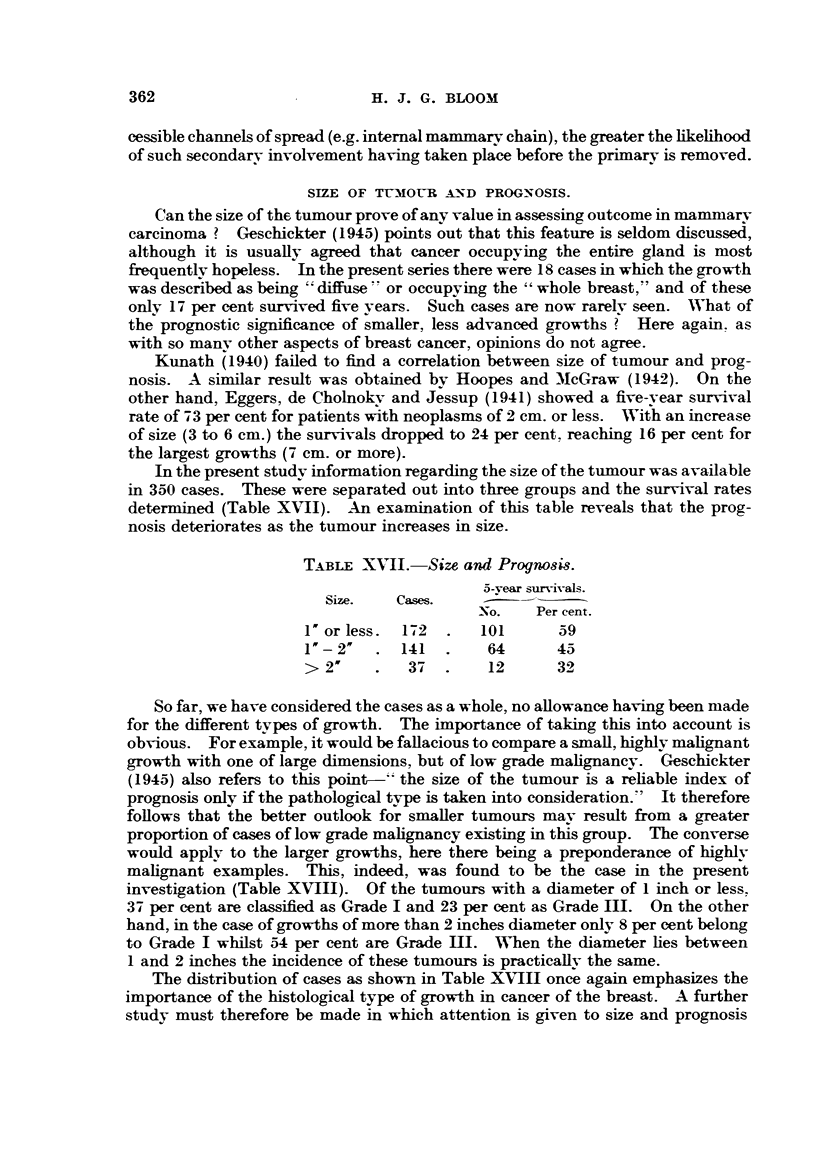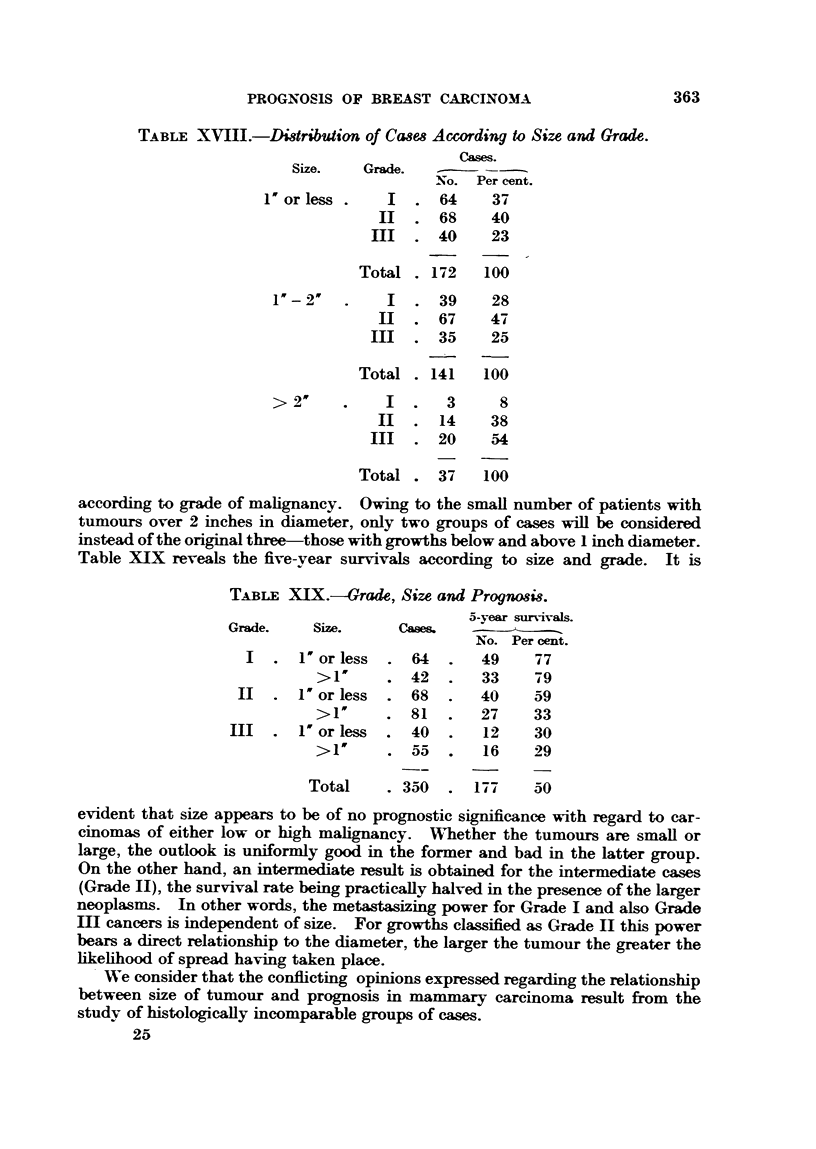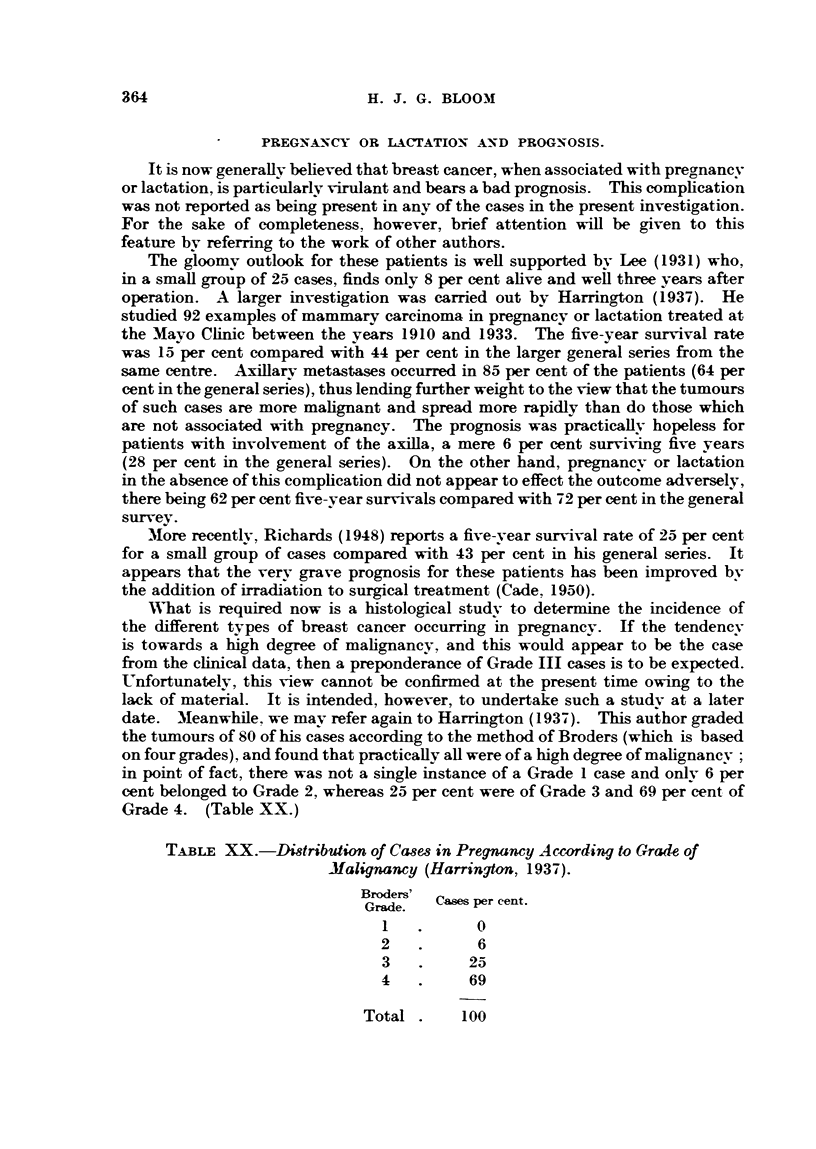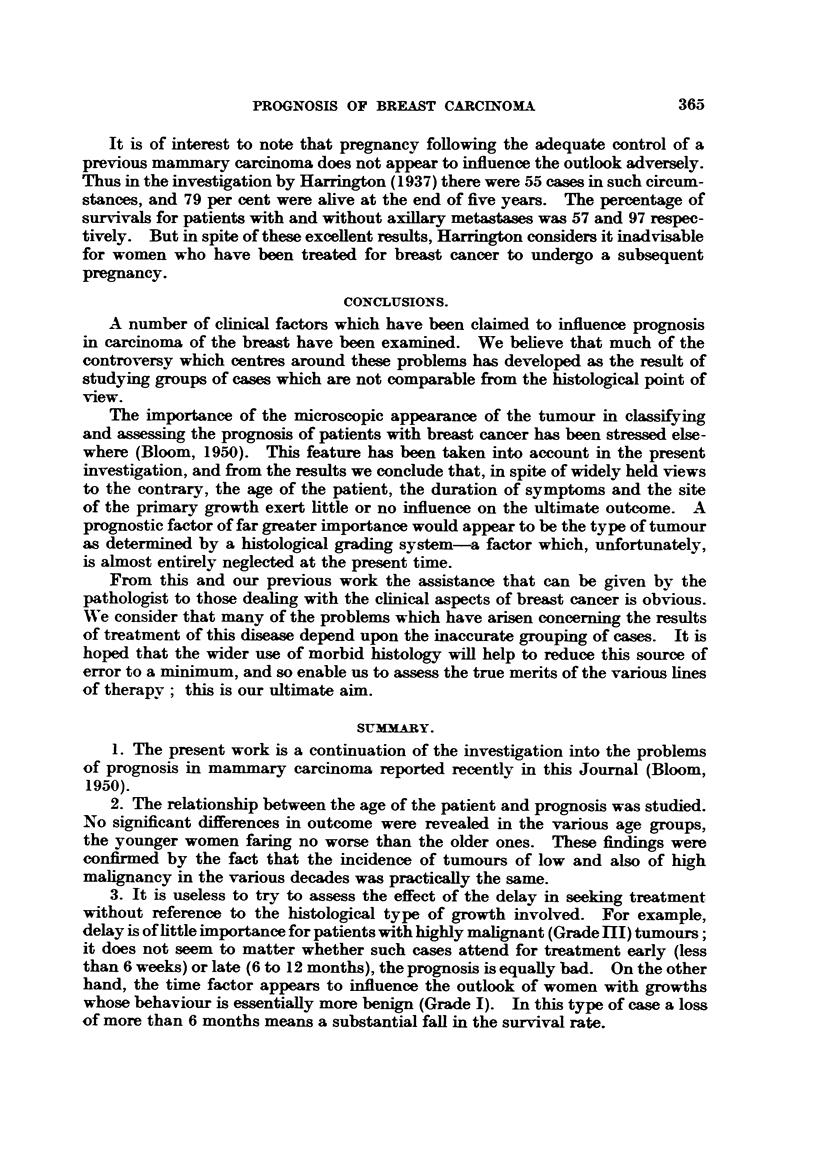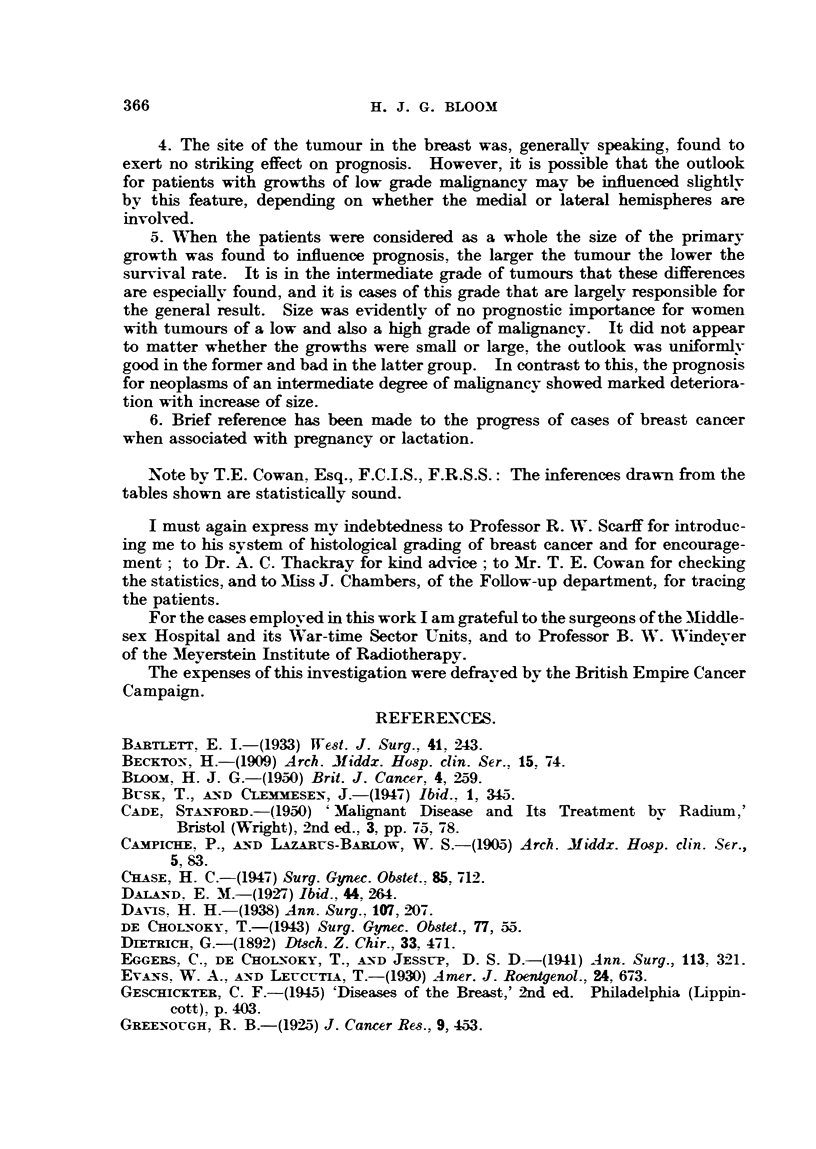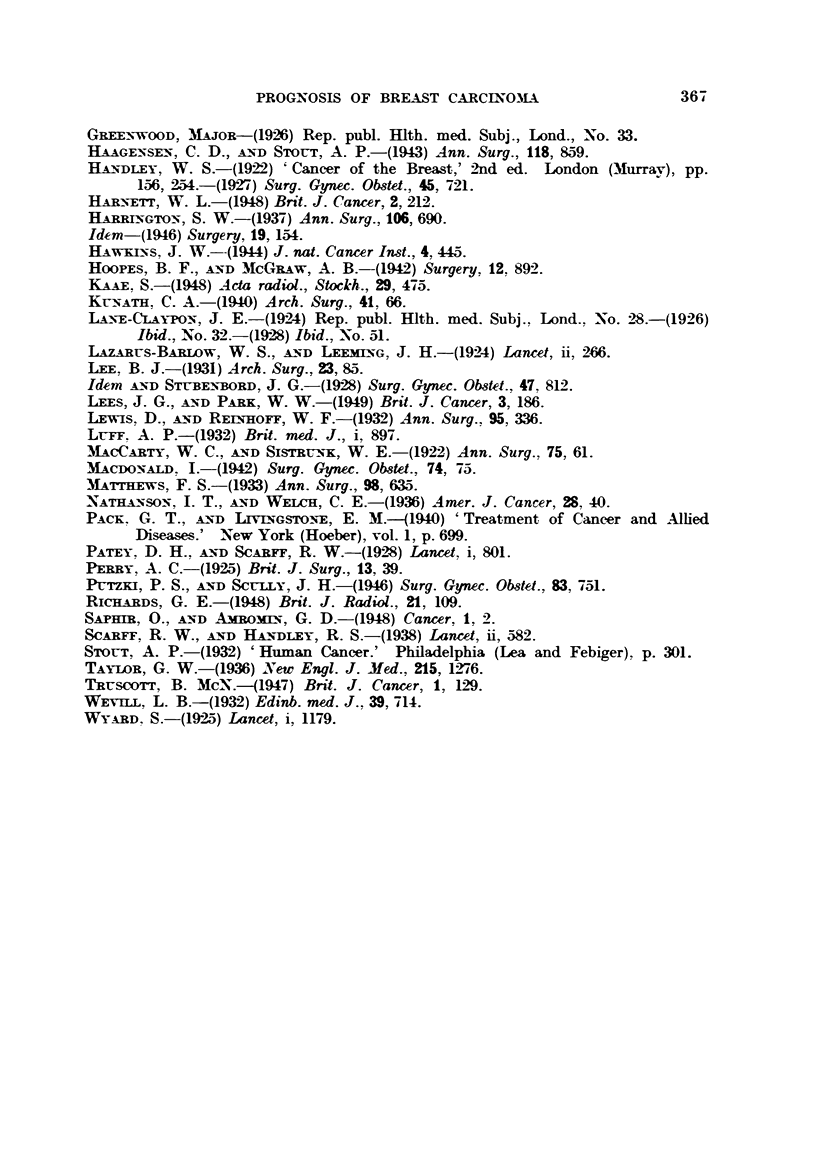# Further Studies on Prognosis of Breast Carcinoma

**DOI:** 10.1038/bjc.1950.34

**Published:** 1950-12

**Authors:** H. J. G. Bloom


BRITISH JOURNAL OF CANCER

VOL. IV DECEMBER, 1950 NO. 4

### FURTHER STUDIES ON PROGNOSIS OF BREAST CARCINOMA.

H. J. G. BLOOM.

*From the Bland-Sutton Institute of Pathology, Middlesex Hospital, London, W.1.*

Received for publication December 8, 1950.

IN a recent communication in this Journal (Bloom, 1950), the significance of the histological architecture of the tumour in cases of mammary carcinoma was investigated. By employing a simple system of grading a close relationship was revealed between histology and prognosis.

A classification based upon the microscopic appearance of the tumour (grade) combined with a knowledge of its extent (stage) was found to give a far more accurate grouping of cases, and also provide a more reliable guide to prognosis than could be achieved by either a system of grading or staging alone. Furthermore, by considering the cases according to our clinico-pathological subdivisions, light was thrown upon some hitherto unexplained problems concerning the results of treatment.

The present paper is a continuation of the above work. It is the object now to investigate some further aspects of prognosis in breast cancer on which, from time to time, widely differing opinions have been expressed. What are these problems, and why have they given rise to confusion ?

Is the prognosis in carcinoma of the breast influenced by the age of the patient ? What is the effect on prognosis of delay in seeking treatment ? Has the size and also the site of the primary growth any bearing on outcome ? These questions will be studied presently, and the significance of the histological grade of malignancy of the tumour will be considered in relation to them.

### *Material.*

The same series of cases employed in our first communication will be used for the present enquiry. It is made up of 565 cases of breast cancer treated at the Middlesex Hospital during the years 1936 to 1942, and at the associated War Sector Units during the latter half of this period. From these patients 95 must be excluded for various reasons already considered (Bloom, 1950) thus leaving us with 470 available for the investigation.

### *Method of Grading.*

The system of histological grading introduced into this country by Patey and Scarff (1928) will be used. This is based upon the principles formulated by

Greenough (1925) in America, but chief importance is attached to the degree of tubule formation, regularity in size and staining of nuclei, and the number of hyperchromatic and mitotic figures. Three classes of tumour are recognized and designated Grade I, Grade II and Grade III, thereby indicating carcinomata of low, intermediate and high degrees of malignancy, respectively. Further details of this method of grading may be found in our previous report (Bloom, 1950), the system proving both simple to apply and remarkably effective in determining possible outcome. The five-year survival rates for the three classes of tumour were as follows : Grade I, 79 per cent ; Grade II, 42 per cent, and Grade III, 25 per cent.

*Assessment of Prognosis.*

The prognostic yard-stick will be the five-years' survival rate. It must be pointed out that " survival rate " does not necessarily imply freedom from cancer ; it is merely an indication of the number of patients actually alive.

### AGE AND PROGNOSIS.

Many authors consider age to have an important bearing on prognosis in cancer of the breast. Lee and Stubenbord (1928) have included a factor for age in their clinical classification which has been employed recently by Richards (1948).

It is widely believed that the younger the patient with malignant disease, the more gloomy the outlook. In the case of mammary carcinoma this view is held by MacCarty and Sistrunk (1922), Perry (1925), Daland (1927), Stout (1932), Nathanson and Welch (1936), Kunath (1940), Cade (1940) and Pack and Livingstone (1940).

Breast cancer occurring before the age of 30 is often regarded as being extremely fatal, and Haagansen and Stout (1943) point out that some surgeons go so far as to decline to operate upon such cases. Lee (1931) made a special study of this disease in young women. He investigated 191 cases treated by radical surgery with or without ancillary radiotherapy ; all were under the age of 40. The three-year survival rate was 40 per cent, and only 16 per cent were without obvious metastases. The author concluded that " cancer of the breast in young women is a much more menacing disease than it is in mid-life or in old age." It is true that these results may be poor for such a short period of follow-up, but attention must be drawn to the fact that no control series of older patients treated at the same clinic was available for comparison. In addition, when the survival rate was determined for various age groups under 40 the results were found to be practically identical. Furthermore, there were 25 cases of lactation cancer in the series, and these should have been considered separately as they represent a special, well-recognized problem. The effects of pregnancy or lactation on breast cancer will be referred to later.

At the Middlesex Hospital, Lazarus-Barlow and Leeming (1924) made their classical study on the natural duration of life for cancer of various organs. With regard to the breast, 243 cases, seen between the years 1883 and 1922, were available for study. The authors revealed a parallel between age and outcome, the mean duration of life becoming greater with advancing years.

We must now turn and consider the opposite view, that age is of no prognostic significance. de Cholnoky (1943) reported the findings in a series of cases, all under the age of 30. Fifty-nine patients were treated by a radical mastectomy with a five-and ten-year survival rate of 41 per cent and 37 per cent respectively. These results are relatively good. In fact, they are even superior to those obtained in a general series studied by the same author—34 per cent five-year and 22 per cent ten-year survival rates (Eggers, de Cholnoky and Jessup, 1941).

Hawkins (1944), in an investigation of some 2,600 cases showed a comparable five-year survival rate in three age groups, namely, 49 and less, 50 to 59 and over 60. The author concluded that these findings do not support the frequent contention that the younger cancer patients have a worse outlook than the older groups.

Age was not found to be an important factor in prognosis by Wyard (1925), Greenwood (1926), Evans and Leucutia (1930), Lewis and Reinhoff (1932), Matthews (1933), Scarff and Handley (1938), Macdonald (1942), Hoopes and McGraw (1942), Haagensen and Stout (1943), Truscott (1947) and Harnett (1948).

Lane-Claypon (1924) in her survey for the Ministry of Health demonstrated a similar survival rate among some 500 cases classified according to decade. In a further paper (Lane-Claypon, 1928) some 1,800 cases were divided into three age groups—less than 40, 40 to 50, and 60 and over. From a study of the survivals for each group, the author concluded that " the statement commonly made that the prognosis is worse in younger persons is erroneous."

More recently, Harrington (1946) in a large series of cases from the Mayo Clinic found no relationship between age and the number of survivals at five years. In addition, the effect of age on the incidence of axillary metastases was investigated. The proportion of cases with this complication were found to be comparable in the majority of groups. Similarly, 59 per cent of the young patients reported by de Cholnoky (1943) had axillary involvement compared with 64 per cent in the same author's general series (Eggers, de Cholnoky and Jessup, 1941).

Can a relationship between age and prognosis be shown in the present series ? The age was available in 461 cases. The youngest patient was 22 and the oldest 80. Table I displays the five-year survivals in the various decades. The results of a broader grouping are also presented (Table II).

If we examine Table I it is evident that there is considerable fluctuation in the percentage of survivals in the different groups. No parallel is revealed

TABLE I.—*Age and Prognosis.*

| Age. | Cases. | 5-year survivals. No. | 5-year survivals. Per cent. |
|---|---|---|---|
| <40 | 45 | 19 | 42 |
| 40–49 | 145 | 84 | 58 |
| 50–59 | 136 | 66 | 49 |
| 60–69 | 87 | 33 | 38 |
| 70+ | 48 | 24 | 50 |

between age and outcome, and there is no support for the view that the tumours of younger women, apart from those associated with pregnancy and lactation, kill more rapidly. Of patients aged 39 or less, 42 per cent were alive at five years compared with 38 per cent in the seventh decade. When the cases are separated out, as in Table II, it is clear again that the younger patients fare no worse than do the older ones, in fact, if anything, they do rather better. This has also been the finding of Lane-Claypon (1928) and Hawkins (1944).

TABLE II.—*Age and Prognosis.*

| Age. | Cases. | 5-year survivals. No. | Per cent. |
|---|---|---|---|
| <50 | 190 | 103 | 54 |
| 50–59 | 136 | 66 | 49 |
| 60+ | 135 | 57 | 42 |

Geschickter (1945) believes that certain unfavourable features exist for younger women with breast cancer, but that these are compensated for by the fact that such patients, as shown by Kaae (1948), tend to seek medical advice sooner than do older groups. Where the prognosis has been found to be comparable for various ages it may be argued that this factor has come into operation. Hence, it may not be adequate to consider the prognostic significance of age purely from the results of treatment.

Many authors who claim a parallel to exist between age and outcome maintain that the breast cancers of younger women tend to be more anaplastic and grow more rapidly than do those in older women. If this is true one would expect to find a greater proportion of growths of high grade malignancy among the younger cases, and a preponderance of low grade tumours in the older groups. Such a discovery would be conclusive evidence for the view that the younger the patient the graver the outlook. Now, several workers have shown that the degree of malignancy in mammary carcinoma can be ascertained by examining a section of the tumour. Attempts, however, to correlate the histology of cancer with age appear to have been rarely performed. Up to the present time only two investigations of this type have been found in the literature (Taylor, 1936 ; Lees and Park, 1949). A study on these lines will now be made.

TABLE III.—*Age, Grade and Prognosis.*

| Age. | Grade. | Cases. No. | Per cent. |
|---|---|---|---|
| <40 | I | 10 | 22·2 |
| | II | 22 | |
| | III | 13 | 28·9 |
| 40–49 | I | 45 | 31·0 |
| | II | 58 | |
| | III | 42 | 29·0 |
| 50–59 | I | 39 | 28·7 |
| | II | 59 | |
| | III | 38 | 27·9 |
| 60–69 | I | 26 | 29·9 |
| | II | 37 | |
| | III | 24 | 27·6 |
| 70+ | I | 16 | 33·3 |
| | II | 16 | |
| | III | 16 | 33·3 |

Table III has been constructed to show the relationship between age and grade of malignancy in breast cancer. An examination of this table reveals a really remarkably even distribution of tumours of low and high grade malignancy in the various decades. Not only is the proportion of Grade I and also of Grade III cases similar in the different groups, but in addition the incidence of these tumours is practically identical in the individual groups. Thus we can find no support whatsoever in the present study for the thesis that cancer of the breast is more malignant in younger than it is in older patients. These results, however, are not in agreement with those obtained by Taylor (1936) who also employed the principles of grading laid down by Greenough (1925). He found a smaller number of low and a greater number of high grade tumours among younger women when compared with an older group. On the other hand, the conclusions reached by Lees and Park (1949), who have recently investigated the relationship between histology and age for cancer in various organs including the breast, agree with our findings and not with those of Taylor (1936).

### DURATION OF SYMPTOMS AND PROGNOSIS.

Generally speaking, the delay in seeking medical advice after the onset of symptoms in breast cancer is becoming shorter. Towards the end of the last century we find Dietrich (1892) reporting only 23 per cent of cases in a series as having symptoms lasting less than six months. Reports from many sources indicate that the proportion of patients attending hospital within this time has gradually increased over the course of the last fifty years. For example, according to Harrington (1946) 33 per cent of the cases seen at the Mayo Clinic between the years 1910 and 1914 were operated upon within six months and 54 per cent within one year of the first symptom. These percentages climbed steadily, reaching 50 and 70 respectively for the period 1935 to 1939.

In the present study data regarding the length of the history were available in 406 cases. Of these, 65 per cent came for treatment within six months of the first symptom and 85 per cent within one year. Similar percentages are given by Kaae (1948). In spite of the improvement, compared with earlier investigations, these figures are still far from satisfactory. On the other hand, reports from elsewhere are much more gloomy. Lewis and Reinhoff (1932) find as few as 34 per cent of their cases seeking medical advice within six months and 63 per cent within one year. Even more recently, Eggers, de Cholnoky and Jessup (1941) quote only 37 per cent and 55 per cent for corresponding intervals of time.

The symptoms of mammary carcinoma and their frequency are dealt with adequately by many authors. Suffice it here to say that by far the commonest initial symptom has always been a lump in the breast. Forty-five years ago Campiche and Lazarus-Barlow (1905) made a study of nearly 2,000 patients seen at the Middlesex Hospital between the years 1747 and 1903, and found this feature present in 63 per cent. Later, at the same hospital, Beckton (1909) gave an incidence of 70 per cent. More recent authors report even higher figures. Thus, Harnett (1948) and also Cade (1940) find a lump to be the first symptom in 77 per cent of cases, Truscott (1947) in 84 per cent and Putzki and Scully (1946) in 88 per cent. The " duration of symptoms," therefore, in the vast majority of cases refers to the length of time a tumour has been present in the breast—from the time the patient found it to the time she attended the hospital clinic.

The responsibility for prolonged delay before the institution of treatment may be entirely that of the patient, but not always. Medical practitioners are occasionally guilty of wasting time, especially with regard to younger women when cancer is not suspected. Kaae (1948) found that in his series three-quarters of the cases were subjected to adequate therapy within one month and 90 per cent within six months of the first medical examination. Lane-Claypon (1926) reported a mean interval of five to six months between the consultation and operation among 670 cases.

Whenever the management of malignant disease is discussed the importance of early diagnosis and early treatment are emphasized. In the case of mammary carcinoma, has the duration of symptoms any bearing on prognosis ? The general teaching has certainly always been that the greater the delay in seeking treatment in this disease the worse the outlook. The impression is also frequently given that if a patient receives early therapy the prognosis is necessarily good. These views meet with almost universal acceptance, and only very rarely have they been questioned. Are they in point of fact well-founded ? For if they are, we should direct more attention to reducing the tragically large numbers of women who, through fear or ignorance, fail to seek medical care until many months have elapsed following the discovery of a lump in the breast. If we can accomplish this, will the number of survivals at five and ten years after operation increase ? Many authorities believe they will.

Let us now consider the opinions which have been expressed regarding the duration of symptoms and prognosis. Luff (1932), in an investigation of over 1,500 cases for the British Medical Association, found that the longer the delay between the first symptom and operation the more gloomy the outlook, as shown by the survival, recurrence and mortality rates (Table IV). A study of this table reveals that with a delay of twelve months the survival rate is halved and the recurrence rate more than doubled. Cade (1950) refers to Luff's work and states that " the mortality of cancer of the breast in England and Wales could be reduced from 7,000 yearly to 1,000, if all cases were adequately treated in the first month of the appearance of the disease."

TABLE IV.—*Prognosis and Duration of Symptoms* (*Luff*, 1932).

| Delay in months. | 4-year survival rate. Cases per cent. | Post-operative recurrence. Cases per cent. | Mortality rate. Cases per cent. |
|---|---|---|---|
| $< 1$ | 31 | 14 | 5 |
| 1–3 | 29 | 14 | 17 |
| 3–6 | 24 | 27 | 16 |
| 6–12 | 24 | 27 | 26 |
| $> 12$ | 16 | 35 | 37 |

Davis (1938), after studying a small series of 75 cases, considers that one of the most important factors in determining prognosis is the delay in treatment. He states that early diagnosis and therapy are essential in the ' cure ' of cancer of the breast, and raises the question of advisability of surgery for patients with a long history.

Hoopes and McGraw (1942) found that the percentage of five-year survivors diminished slightly with increase in duration of symptoms, regardless of whether or not the axillary glands were invaded.

Macdonald (1942), in a large series of cases, revealed that the greatest proportion of five-year survivals were among those treated within two months of the tumour being discovered. After this period a rapid deterioration in results occurred. With a delay of one year, however, it was surprising to find the numbers alive increasing. Similar results were obtained by Eggers, de Cholnoky and Jessup (1941), who report a five-year survival rate of 76 per cent for patients operated upon within one month. Here again, the results deteriorated rapidly with increasing duration of symptoms, falling to 20 per cent for a delay of one to two years. With further loss of time, however, the results improved, from 25 per cent for a lapse of two to three years to 41 per cent for an even greater interval.

From the preceding data it would appear that duration of symptoms has a direct bearing on prognosis—the longer they have been present the worse the outlook. The significance of the increase in the survival rates after a delay of one or more years, as found by Macdonald (1942) and also by Eggers, de Cholnoky and Jessup (1941), will be discussed later.

What can be revealed in the present series of cases as to the relationship between delay in seeking treatment and prognosis ? Information regarding this point was available in 406 instances. The five-year survivals were determined according to the duration of symptoms, and these are displayed in Table V. It

TABLE V.—*Duration of Symptoms and Prognosis.*

| Duration of symptoms. | Cases. | 5-year survivals. No. | 5-year survivals. Per cent. |
|---|---|---|---|
| 6 weeks or less | 101 | 50 | 50 |
| 3 months or less | 184 | 94 | 51 |
| 3–6 months | 125 | 59 | 47 |
| 6–12 months | 118 | 56 | 47 |
| 12 months or more | 105 | 55 | 52 |

is to be noted that some overlap occurs in the various groups. Examination of this table shows a truly remarkable state of affairs. The survival rate is uniform, no matter how long the history. We are not able, therefore, to agree with the findings of Luff (1932), Davis (1938), Eggers, de Cholnoky and Jessup (1941), Hoopes and McGraw (1942) and Macdonald (1942). Our results also appear to conflict with generally accepted views, but they are not unique. Lewis and Rienhoff (1932) in 950 cases, Hawkins (1944) in over 3,000 cases and Kunath (1940) consider that duration of symptoms, *per se*, are of little prognostic significance.

This problem must be now examined more closely. So far, we have studied the cases as a whole. No attempt has been made to classify them, and the importance of this procedure has been stressed elsewhere (Bloom, 1950). What types of cases are present in the various groups shown in Table V? It is possible that these groups are not comparable. For example, there may be a preponderance of highly malignant tumours among the women attending hospital within the six-week period, whilst there exists but a small proportion of similar growths among those delaying for one year or longer. Such a distribution may compensate for the difference in time, and thus account for the practically identical end-results.

Cases of breast cancer may be classified by clinical stage or histological grade.

Let us first of all consider the scatter of the patients according to stage and duration of symptoms. For this purpose the Manchester system was employed, a summary of which may be found in our previous report (Bloom, 1950). Although this classification depends upon clinical data, glandular involvement was determined, where possible, by microscopic examination ; the reasons for this have been dealt with in the same communication.

Reference to Table VI shows that with increasing length of history there is a progressive fall in the proportion of cases at an early stage (Stage 1). A similar

TABLE VI.—*Distribution of cases according to Duration of Symptoms and Clinical Stage.*

| Duration of symptoms. | Total cases. | Cases in Stage 1. Per cent. | Cases in Stage 2. Per cent. | Cases in Stage 3. Per cent. | Cases in Stage 4. Per cent. | Total. Per cent. |
|---|---|---|---|---|---|---|
| 6 weeks or less | 101 | 39 | 38 | 19 | 4 | 100 |
| 3 months or less | 184 | 37 | 39 | 19 | 5 | 100 |
| 3 to 6 months | 125 | 35 | 34 | 23 | 8 | 100 |
| 6 to 12 months | 118 | 33 | 30 | 31 | 6 | 100 |
| 1 year or more | 105 | 25 | 21 | 44 | 10 | 100 |

state of affairs is seen for those in Stage 2. On the other hand, the advanced cases (Stages 3 and 4) increase in number with prolonged delay. In other words, as more and more time elapses from the onset of the disease, as determined by the patient, so the early and moderately advanced tumours fall in number whilst the proportion of more extensive growths becomes greater.

These results deserve no special comment. They are what one expects and support the conclusions of Kaae (1948) that " with increasing duration of symptoms the number of Stage 1 cases becomes steadily less and there is a gradual increase in the number of inoperables. . . ." On the other hand, these findings make it even more difficult to understand those shown in Table V. For instance, the percentage of Stage 1 and Stage 3 cases among the patients presenting within six weeks of the first symptom is 39 per cent and 19 per cent respectively (Table VI). After a delay of one year or more the proportion of early cases falls to 25 per cent, whilst the advanced cases increase to 44 per cent. And yet, in spite of this, the five-year survival rate is practically the same—50 per cent for those having a delay of six weeks or less compared with 52 per cent for those waiting one year or longer (Table V).

Will a study of the histological type of tumour throw any light on the problem ? The distribution of cases according to duration of symptoms and grade have been drawn out in Table VII. A comparable percentage of Grade I tumours is seen

TABLE VII.—*Distribution of Cases According to Duration of Symptoms and Histological Grade.*

| Duration of symptoms. | Total cases. | Cases in Grade I. Per cent. | Cases in Grade II. Per cent. | Cases in Grade III. Per cent. | Total. Per cent. |
|---|---|---|---|---|---|
| 6 weeks or less | 101 | 24 | 45 | 31 | 100 |
| 3 months or less | 184 | 25 | 45 | 30 | 100 |
| 3 to 6 months | 125 | 23 | 47 | 30 | 100 |
| 6 to 12 months | 118 | 33 | 40 | 27 | 100 |
| 1 year or more | 105 | 38 | 34 | 28 | 100 |

among the patients attending hospital within six weeks, three months and three to six months of the first symptom. A similar state of affairs exists for Grade II and also Grade III carcinomas. After this time, however, there is an increase in the proportion of Grade I cases, and a reduction in those belonging to the higher grades of malignancy. These changes, although not marked, may indicate that the more virulent tumours tend to present sooner than the ones of lower malignancy.

Let us investigate this matter further by considering the delay in seeking treatment of the cases according to the grade of tumour. Table VIII has been

TABLE VIII.—*Percentage of Cases in Each Histological Grade According to Duration of Symptoms.*

| Grade. | Duration of symptoms in months. 6 or less. Cases per cent. | 7 to 12. Cases per cent. | More than 12. Cases per cent. | Total per cent. |
|---|---|---|---|---|
| I | 56 | 21 | 23 | 100 |
| II | 70 | 18 | 12 | 100 |
| III | 69 | 21 | 10 | 100 |

constructed on a broader basis than Table VII, three periods only being taken, namely, less than six months, between seven and twelve months and more than one year. These results lend more weight to the view that patients with tumours of high malignancy tend to consult their doctors earlier than do those with growths of low malignancy. In addition, the mean duration of symptoms for the three classes of tumour are presented in Table IX. Here again there is evidence that

TABLE IX.—*Mean Duration of Symptoms According to Grade.*

| Grade. | Cases. | Mean duration of symptoms in months. |
|---|---|---|
| I | 124 | 10.1 |
| II | 174 | 7.6 |
| III | 125 | 7.1 |
| Total | 423 | 8.3 |

the most malignant neoplasms are associated with the shortest histories. This, presumably, depends upon the rate of growth of the cancer, the higher grades growing more rapidly. Thus, larger tumours are discovered more easily, and a rapid increase in size alarms the patient to seek medical advice at an early date. The small, slowly growing tumours of low grade malignancy may be regarded by patients, at first, as not being serious, and so more time elapses before the medical consultation.

Let us summarize briefly what has been said so far : Opposing views regarding the effect of delay in seeking treatment on prognosis have been considered. From our own series we have not been able to show, in spite of the findings in Table VI that outlook becomes more gloomy with increasing delay. It was only when the histological grade of malignancy was studied that some light was thrown on the problem. It appears that the distribution of the types of carcinoma compensate to some extent for the time factor. With regard to the reports of other workers, it may well be that the conflicting results obtained depend upon the comparison of histologically incomparable groups of cases.

In view of these findings let us once again consider the relationship between duration of symptoms and prognosis, but this time according to the grade of malignancy (Table X). It is evident that outcome is now shown, in certain cases,

TABLE X.—*Duration of Symptoms and Prognosis According to Histological Grade.*

| Duration of symptoms. | Grade I. Cases. | Grade I. Alive at 5 years. Per cent. | Grade II. Cases. | Grade II. Alive at 5 years. Per cent. | Grade III. Cases. | Grade III. Alive at 5 years. Per cent. |
|---|---|---|---|---|---|---|
| 6 weeks or less | 24 | 92 | 46 | 41 | 31 | 29 |
| 3 months or less | 45 | 87 | 83 | 47 | 56 | 29 |
| 3 to 6 months | 28 | 86 | 59 | 42 | 38 | 26 |
| 6 to 12 months | 39 | 72 | 47 | 43 | 32 | 25 |
| 1 year or more | 40 | 78 | 36 | 50 | 29 | 21 |

to be influenced by the length of history. Examination of this table in greater detail allows the following conclusions to be reached. The delay in seeking medical advice appears to be of some importance for Grade I cases ; of these attending hospital within six weeks of the first symptom, 92 per cent survive five years, compared with 72 per cent waiting six to twelve months. In contrast, there is little difference in outlook for patients with Grade II tumours for similar intervals of time. In the case of the highest grade of carcinoma there is a negligible fall in results with increasing delay. The survival rate in this group is 29 per cent for patients seeking treatment within six weeks of the onset, compared with 25 per cent for those waiting six to twelve months. We are thus faced with the fact that by the time a highly malignant growth (Grade III) is discovered by the patient it is, in all probability, too late to eradicate, direct extension and metastasis having already taken place.

It must be pointed out, however, that many women with Grade III cancers who present early appear, from the clinical aspect, to have a favourable outlook. Why then is disaster so frequent in these cases ? The theory is held here that such cases, in reality, are sub-clinically advanced, small tumour emboli probably having already reached regional glands and bone. Such deposits have not yet had time to grow and become evident. Even routine histological examination of axillary glands removed by the radical mastectomy may fail to reveal their presence. Saphir and Amromin (1948), by serial sections, have demonstrated cancer cells in the axillary glands in about one-third of cases previously reported as being free from invasion.

We are compelled to adopt the view that outcome in mammary carcinoma is determined largely by the histological type of growth, rather than by prompt treatment as soon as the lesion is discovered. Macdonald (1942) expresses a similar opinion when he states that " natural selection " is more important in prognosis than early therapy. Kunath (1940) considers that " the rate of growth " is the major factor influencing end-results rather than the length of time the tumour has been present. In fact, Nathanson and Welch (1936) believe that patients with the shortest delay in treatment have the worst prognosis ! This unorthodox view is based upon evidence which suggests that patients with tumours of slow growth tend to seek medical aid much later than those with ones of rapid growth ; some support for this has been found in Tables VIII and IX.

Certain authors have shown a sudden increase in the five-year results when the duration of symptoms reaches one year or longer. This has been the experience of Eggers, de Cholnoky and Jessup (1941), Macdonald (1942) and Harnett (1948). In the present series no such increase was observed when the patients were taken as a whole (Table V). On the other hand, a slight improvement was demonstrated by considering the cases according to histological grade (Table X). This only applied to the carcinomas belonging to the low and intermediate grades of malignancy. It is postulated that these tumours of long duration were of particularly slow growth and, therefore, did not alarm the patients sufficiently to visit their doctors until after a considerable length of time had elapsed.

What conclusions can be reached here regarding the duration of symptoms and prognosis in breast cancer? Pleas are for ever being made for the education of the public and general practitioner with a view to shortening the delay in seeking treatment after the onset of the first symptom. This may appear to be justifiable when it is remembered that, even in modern communities subject to press and radio propaganda, there are still, at the best, only some 60 per cent of patients who present within six months of discovering a lump in the breast. Lost time, however, has been shown here to be of importance only for tumours of low grade malignancy. With regard to the most virulent growths (Grade III) this factor appears to be of little or no significance. The theory is put forward that by the time such a tumour is discovered by the patient it has already spread beyond the breast.

Perhaps we may now refer briefly to certain clinical implications arising from our investigation. What can be done to improve the prognosis of the Grade III cases ? It would appear that the favourable stage for the treatment of this type of growth is long before it is found by the patient. Frequent routine examinations of the breasts would, in all probability, lead to its early discovery, and perhaps a better chance for attaining a five-year survival. Several surgeons have suggested such a plan. Indeed, Hawkins (1944) has gone so far as to advocate that every woman should be taught to inspect and palpate her own breasts, and that such an examination be made not less than once a month. Chase (1947) voices a similar opinion. When it is recalled that about 7,000 women in this country and 15,000 in the United States of America die annually from breast cancer, some may consider this procedure to be warranted. Others, for fear of producing a widespread cancer neurosis, would rather direct energy to improving the treatment of the disease.

Doubt has been expressed as to the value of surgical treatment, purely on account of a long history (Davis, 1938). From the evidence presented in Table X it would appear that such a view is not only fallacious, but also extremely dangerous ; of the patients with symptoms lasting one year or more, over three-quarters of those belonging to Grade I and fully a half of those of Grade II were alive five years after operation.

In conclusion : we believe that campaigns aimed at shortening the delay before seeking medical advice should not only be continued, but also intensified, if only for the sake of those patients having tumours of low histological malignancy.

It is most important, however, to realize that such measures alone must not be relied upon to increase the number of five-and ten-year survivors. We have revealed that the prognosis of certain cases does not appear to be materially influenced by the institution of early therapy. In fact, it is misleading to state, as so often is done, that the outlook is usually good for patients who present promptly after the onset of symptoms.

### SITE OF TUMOUR AND PROGNOSIS.

Before investigating the relationship between site of growth and prognosis in breast carcinoma, let us first examine the frequency with which various parts of the gland are involved by this disease.

The incidence of cancer in either breast appears to be about equal. A number of reports, however, suggest a slight preponderance, to the extent of 4 or 5 per cent, for the left side over the right (Luff, 1932 ; Wevill, 1932 ; Busk and Clemmesen, 1947 ; Harnett, 1948).

The region of the breast affected was studied in the present series, the usual subdivisions into quadrants, axillary tail and centre being made. Adequate information regarding the site of the growth was available in 441 cases. In 52 of these the tumour was described as being situated in a hemisphere, or in the mid-line of two adjacent quadrants. Four patients were said to have more than one lump in different quadrants of a single breast. It was thought best to exclude these 56 cases from the series, leaving 385 for consideration. The distribution of the tumours, which is drawn out in Table XI, reveals a well-marked preponderance of the upper outer quadrant of the breast as the site for mammary cancer.

TABLE XI.—*Incidence of Breast Carcinoma According to Site (Middlesex Hospital).*

| Site. | Present series (1936 to 1942) 385 cases. | | Campiche and Barlow, 1905 (1747 to 1903) 1010 cases. | Beckton, 1909 (1904 to 1909) 230 cases. | Truscott, 1947 (1926 to 1935) 836 cases. |
|---|---|---|---|---|---|
| | Cases. | Per cent. | Per cent. | Per cent. | Per cent. |
| Upper outer | 177 | 46 | 45 | 46 | 46 |
| Upper inner | 75 | 19 | 17 | 20 | 20 |
| Central | 35 | 9 | 20 | 15 | 13 |
| Lower outer | 54 | 14 | 12 | 13 | 12 |
| Lower inner | 19 | 5 | 6 | 5 | 5 |
| Axillary tail | 7 | 2 | – | 1 | 4 |
| Diffuse or whole breast | 18 | 5 | – | – | – |
| Total | 385 | 100 | 100 | 100 | 100 |

The frequencies given here for the different regions agree very closely with those of previous workers at the Middlesex Hospital, also shown in Table XI. The somewhat higher incidence of "central" growths found by Campiche and Lazarus-Barlow (1905), Beckton (1909) and Truscott (1947) is probably accounted for by the inclusion in this group of large growths involving three or more quadrants. We have preferred to classify these separately under the heading of "diffuse" or "whole breast." The results obtained at this hospital differ in no way from those reported elsewhere (Lane-Claypon, 1924 ; Luff, 1932 ; Wevill, 1932 ; Geschickter, 1945 ; Harnett, 1948).

Having noted the frequency with which the various parts of the breast are the seat of cancer, we will now consider whether these sites have any prognostic significance. Many opinions have been expressed, but there is no general agreement on this point. Handley (1922, 1927) was the first to stress the importance of the internal mammary chain of lymph glands as an avenue of extension for malignant cells to the thorax and abdomen. Because of the proximity to these surgically inaccessible nodes, carcinomas arising in the inner half of the breast are alleged by some authors, such as Bartlett (1933) and Hawkins (1944), to carry a more gloomy prognosis than those situated in the outer hemisphere.

It has also been stated that cancer in the upper outer or lower inner quadrants is more likely to prove fatal at an early date, the former being in close relation to the axillary lymph glands and the latter to the abdominal viscera. A similar opinion has been expressed for tumours in the region of the nipple, owing to the presence of the sub-areolar plexus of Sappey which drains to the axilla. Lane-Claypon (1924) found support for these views in a group of some 300 cases. This same author, however, in a later, more extensive study (Lane-Claypon, 1928) was unable to confirm her previous results, and finally decided that " there is no evidence that the prognosis varies according to the site." More recently, Truscott (1947) and also Harnett (1948) have reached a similar conclusion.

The relationship between site and survival rate was examined in the present series, the results being shown in Table XII. Owing to the very small number of

TABLE XII.—*Site and Prognosis.*

| Site. | Cases. | 5-year survivals. No. | 5-year survivals. Per cent. |
|---|---|---|---|
| Upper outer | 177 | 92 | 52 |
| Lower outer | 54 | 31 | 57 |
| Central | 35 | 15 | 43 |
| Upper inner | 75 | 35 | 47 |
| Lower inner | 19 | 9 | 47 |
| Axillary tail | 7 | 1 | 14 |
| Diffuse or whole breast | 18 | 3 | 17 |
| Total | 385 | 186 | 48 |

examples, we cannot consider the prognostic significance of axillary tail involvement. The cases classified as " diffuse " or " whole breast " appear to have a bad outlook, a mere 17 per cent surviving five years. As for the other sites, there is no striking variation in the results. It is possible that growths situated in the sternal quadrants and in the nipple area carry a slightly worse prognosis than do those in the outer regions.

A broader grouping of cases will now be considered, depending on whether the inner or outer hemisphere of the breast is the seat of the tumour (Table XIII).

TABLE XIII.—*Site and Prognosis.*

| Site. | Cases. | 5-year survivals. No. | 5-year survivals. Per cent. |
|---|---|---|---|
| Inner hemisphere | 94 | 44 | 47 |
| Outer hemisphere | 243 | 130 | 53 |

The survival rate is again seen to be less for the inner region, but the difference is very small and therefore of doubtful significance. Consequently, we have not been able, so far, to prove that tumours situated in the sternal half of the breast carry a more gloomy outlook than those in the axillary half. In fact, a previous writer from this Hospital (Truscott, 1947) found that patients with growths in the lower inner quadrant had the lowest mortality.

When we have been previously faced with conflicting opinions on various aspects of breast cancer, considerable assistance has been obtained by studying the morbid histology of our cases. Let us, therefore, apply the principle of grading to the present problem.

First of all, what types of cases are present in the various groups we have considered in Table XII ? The distribution according to the grade of malignancy is shown for each site in Table XIV.

TABLE XIV.—*Incidence of the Three Grades of Tumour According to the Part of the Breast Involved.*

| Grade. | Cases per cent. | | | | | | |
|---|---|---|---|---|---|---|---|
| | Axillary tail. | Central. | Diffuse. | Quadrant. Upper outer. | Quadrant. Upper inner. | Quadrant. Lower outer. | Quadrant. Lower inner. |
| I | 0 | 37 | 11 | 31 | 30 | 30 | 32 |
| II | 29 | 40 | 28 | 44 | 41 | 40 | 36 |
| III | 71 | 23 | 61 | 25 | 29 | 30 | 32 |
| Total | 100 | 100 | 100 | 100 | 100 | 100 | 100 |

It is evident that the poor prognosis for the small number of patients with axillary tail growths could be entirely accounted for by the preponderance of highly malignant tumours in this group, there being 71 per cent Grade III but no Grade I cases.

A study of those patients classified as having " diffuse " cancers shows a similar state of affairs. In this instance 61 per cent of the patients had Grade III tumours, whilst only 11 per cent were Grade I. The widespread nature of these growths and the poor prognosis would, therefore, appear to depend upon the high incidence of extremely malignant tumours.

Further examination of Table XIV reveals that, in contrast to the " axillary " and " diffuse " tumours, the proportion of the three types of cancer in the central and various quadrant positions of the breast is very similar. Hence, in these cases we cannot evoke the distribution of the tumours to account for the slight differences in the five-year survival rates.

With regard to the cases separated out according to inner and outer hemisphere involvement (Table XIII), the scatter of the cases in each group is again seen to be similar (Table XV).

TABLE XV.—*Incidence of the Three Grades of Tumour According to Inner or Outer Hemisphere Involvement of the Breast.*

| Grade. | Cases per cent. Inner Hemisphere. | Outer Hemisphere. |
|---|---|---|
| I | 31 | 31 |
| II | 41 | 39 |
| III | 28 | 30 |
| Total | 100 | 100 |

Let us now reconsider the problem of site and prognosis, but this time taking into account the grade of malignancy. To avoid breaking the cases up into too many small groups, only inner and outer hemisphere growths will be studied. The results of this investigation are laid out in Table XVI. It is interesting to

TABLE XVI.—*Site, Grade and Prognosis.*

| Grade. | Outer hemisphere. | | | Inner hemisphere. | | |
|---|---|---|---|---|---|---|
| | Cases. | 5-year survivors. | | Cases. | 5-year survivors. | |
| | | No. | Per cent. | | No. | Per cent. |
| I | 76 | 65 | 86 | 29 | 21 | 72 |
| II | 100 | 47 | 47 | 37 | 15 | 41 |
| III | 67 | 18 | 27 | 28 | 8 | 29 |

note that site may be of some importance for Grade I carcinomas, the outlook being slightly worse when the inner half of the breast is involved. The difference for the intermediate cases is very small and of doubtful significance. Tumours of high grade malignancy are seen to have an equally bad prognosis whether they are situated in the inner or outer regions.

We conclude from the present investigation that, generally speaking, site of tumour in breast cancer exerts no striking effect on prognosis, apart from those patients in whom the growth involves a large part of the gland, and this is largely determined by the histological type of neoplasm (i.e. Grade III). On the other hand, the outlook for the women with centrally placed growths may be slightly less favourable than for those with quadrant tumours, presumably because of their close proximity to the sub-areola lymphatic plexus of Sappey. Cases of low grade malignancy may be influenced to a minor degree by the site of the growth, depending upon whether the sternal or axillary half of the breast is affected (Table XVI). The reason for this may be as follows. Site is of no consequence for Grade III tumours owing to their tendency to metastasize early—no matter where they are situated, rapid, wide-spread secondary deposits appear to be the rule. In contrast to this, Grade I tumours disseminate much less readily. Hence site becomes important, the nearer these growths are to inaccessible channels of spread (e.g. internal mammary chain), the greater the likelihood of such secondary involvement having taken place before the primary is removed.

SIZE OF TUMOUR AND PROGNOSIS.

Can the size of the tumour prove of any value in assessing outcome in mammary carcinoma ? Geschickter (1945) points out that this feature is seldom discussed, although it is usually agreed that cancer occupying the entire gland is most frequently hopeless. In the present series there were 18 cases in which the growth was described as being "diffuse" or occupying the "whole breast," and of these only 17 per cent survived five years. Such cases are now rarely seen. What of the prognostic significance of smaller, less advanced growths ? Here again, as with so many other aspects of breast cancer, opinions do not agree.

Kunath (1940) failed to find a correlation between size of tumour and prognosis. A similar result was obtained by Hoopes and McGraw (1942). On the other hand, Eggers, de Cholnoky and Jessup (1941) showed a five-year survival rate of 73 per cent for patients with neoplasms of 2 cm. or less. With an increase of size (3 to 6 cm.) the survivals dropped to 24 per cent, reaching 16 per cent for the largest growths (7 cm. or more).

In the present study information regarding the size of the tumour was available in 350 cases. These were separated out into three groups and the survival rates determined (Table XVII). An examination of this table reveals that the prognosis deteriorates as the tumour increases in size.

TABLE XVII.—*Size and Prognosis.*

| Size. | Cases. | 5-year survivals. | |
|---|---|---|---|
| | | No. | Per cent. |
| 1″ or less | 172 | 101 | 59 |
| 1″ – 2″ | 141 | 64 | 45 |
| > 2″ | 37 | 12 | 32 |

So far, we have considered the cases as a whole, no allowance having been made for the different types of growth. The importance of taking this into account is obvious. For example, it would be fallacious to compare a small, highly malignant growth with one of large dimensions, but of low grade malignancy. Geschickter (1945) also refers to this point—"the size of the tumour is a reliable index of prognosis only if the pathological type is taken into consideration." It therefore follows that the better outlook for smaller tumours may result from a greater proportion of cases of low grade malignancy existing in this group. The converse would apply to the larger growths, here there being a preponderance of highly malignant examples. This, indeed, was found to be the case in the present investigation (Table XVIII). Of the tumours with a diameter of 1 inch or less, 37 per cent are classified as Grade I and 23 per cent as Grade III. On the other hand, in the case of growths of more than 2 inches diameter only 8 per cent belong to Grade I whilst 54 per cent are Grade III. When the diameter lies between 1 and 2 inches the incidence of these tumours is practically the same.

The distribution of cases as shown in Table XVIII once again emphasizes the importance of the histological type of growth in cancer of the breast. A further study must therefore be made in which attention is given to size and prognosis

TABLE XVIII.—*Distribution of Cases According to Size and Grade.*

| Size. | Grade. | Cases. No. | Cases. Per cent. |
|---|---|---|---|
| 1″ or less | I | 64 | 37 |
| | II | 68 | 40 |
| | III | 40 | 23 |
| | Total | 172 | 100 |
| 1″ - 2″ | I | 39 | 28 |
| | II | 67 | 47 |
| | III | 35 | 25 |
| | Total | 141 | 100 |
| > 2″ | I | 3 | 8 |
| | II | 14 | 38 |
| | III | 20 | 54 |
| | Total | 37 | 100 |

according to grade of malignancy. Owing to the small number of patients with tumours over 2 inches in diameter, only two groups of cases will be considered instead of the original three—those with growths below and above 1 inch diameter. Table XIX reveals the five-year survivals according to size and grade. It is

TABLE XIX.—*Grade, Size and Prognosis.*

| Grade. | Size. | Cases. | 5-year survivals. No. | 5-year survivals. Per cent. |
|---|---|---|---|---|
| I | 1″ or less | 64 | 49 | 77 |
| | >1″ | 42 | 33 | 79 |
| II | 1″ or less | 68 | 40 | 59 |
| | >1″ | 81 | 27 | 33 |
| III | 1″ or less | 40 | 12 | 30 |
| | >1″ | 55 | 16 | 29 |
| | Total | 350 | 177 | 50 |

evident that size appears to be of no prognostic significance with regard to carcinomas of either low or high malignancy. Whether the tumours are small or large, the outlook is uniformly good in the former and bad in the latter group. On the other hand, an intermediate result is obtained for the intermediate cases (Grade II), the survival rate being practically halved in the presence of the larger neoplasms. In other words, the metastasizing power for Grade I and also Grade III cancers is independent of size. For growths classified as Grade II this power bears a direct relationship to the diameter, the larger the tumour the greater the likelihood of spread having taken place.

We consider that the conflicting opinions expressed regarding the relationship between size of tumour and prognosis in mammary carcinoma result from the study of histologically incomparable groups of cases.

PREGNANCY OR LACTATION AND PROGNOSIS.

It is now generally believed that breast cancer, when associated with pregnancy or lactation, is particularly virulant and bears a bad prognosis. This complication was not reported as being present in any of the cases in the present investigation. For the sake of completeness, however, brief attention will be given to this feature by referring to the work of other authors.

The gloomy outlook for these patients is well supported by Lee (1931) who, in a small group of 25 cases, finds only 8 per cent alive and well three years after operation. A larger investigation was carried out by Harrington (1937). He studied 92 examples of mammary carcinoma in pregnancy or lactation treated at the Mayo Clinic between the years 1910 and 1933. The five-year survival rate was 15 per cent compared with 44 per cent in the larger general series from the same centre. Axillary metastases occurred in 85 per cent of the patients (64 per cent in the general series), thus lending further weight to the view that the tumours of such cases are more malignant and spread more rapidly than do those which are not associated with pregnancy. The prognosis was practically hopeless for patients with involvement of the axilla, a mere 6 per cent surviving five years (28 per cent in the general series). On the other hand, pregnancy or lactation in the absence of this complication did not appear to effect the outcome adversely, there being 62 per cent five-year survivals compared with 72 per cent in the general survey.

More recently, Richards (1948) reports a five-year survival rate of 25 per cent for a small group of cases compared with 43 per cent in his general series. It appears that the very grave prognosis for these patients has been improved by the addition of irradiation to surgical treatment (Cade, 1950).

What is required now is a histological study to determine the incidence of the different types of breast cancer occurring in pregnancy. If the tendency is towards a high degree of malignancy, and this would appear to be the case from the clinical data, then a preponderance of Grade III cases is to be expected. Unfortunately, this view cannot be confirmed at the present time owing to the lack of material. It is intended, however, to undertake such a study at a later date. Meanwhile, we may refer again to Harrington (1937). This author graded the tumours of 80 of his cases according to the method of Broders (which is based on four grades), and found that practically all were of a high degree of malignancy ; in point of fact, there was not a single instance of a Grade 1 case and only 6 per cent belonged to Grade 2, whereas 25 per cent were of Grade 3 and 69 per cent of Grade 4. (Table XX.)

TABLE XX.—*Distribution of Cases in Pregnancy According to Grade of Malignancy* (*Harrington*, 1937).

| Broders' Grade. | Cases per cent. |
|---|---|
| 1 . | 0 |
| 2 . | 6 |
| 3 . | 25 |
| 4 . | 69 |
| Total . | 100 |

It is of interest to note that pregnancy following the adequate control of a previous mammary carcinoma does not appear to influence the outlook adversely. Thus in the investigation by Harrington (1937) there were 55 cases in such circumstances, and 79 per cent were alive at the end of five years. The percentage of survivals for patients with and without axillary metastases was 57 and 97 respectively. But in spite of these excellent results, Harrington considers it inadvisable for women who have been treated for breast cancer to undergo a subsequent pregnancy.

### CONCLUSIONS.

A number of clinical factors which have been claimed to influence prognosis in carcinoma of the breast have been examined. We believe that much of the controversy which centres around these problems has developed as the result of studying groups of cases which are not comparable from the histological point of view.

The importance of the microscopic appearance of the tumour in classifying and assessing the prognosis of patients with breast cancer has been stressed elsewhere (Bloom, 1950). This feature has been taken into account in the present investigation, and from the results we conclude that, in spite of widely held views to the contrary, the age of the patient, the duration of symptoms and the site of the primary growth exert little or no influence on the ultimate outcome. A prognostic factor of far greater importance would appear to be the type of tumour as determined by a histological grading system—a factor which, unfortunately, is almost entirely neglected at the present time.

From this and our previous work the assistance that can be given by the pathologist to those dealing with the clinical aspects of breast cancer is obvious. We consider that many of the problems which have arisen concerning the results of treatment of this disease depend upon the inaccurate grouping of cases. It is hoped that the wider use of morbid histology will help to reduce this source of error to a minimum, and so enable us to assess the true merits of the various lines of therapy ; this is our ultimate aim.

### SUMMARY.

1. The present work is a continuation of the investigation into the problems of prognosis in mammary carcinoma reported recently in this Journal (Bloom, 1950).

2. The relationship between the age of the patient and prognosis was studied. No significant differences in outcome were revealed in the various age groups, the younger women faring no worse than the older ones. These findings were confirmed by the fact that the incidence of tumours of low and also of high malignancy in the various decades was practically the same.

3. It is useless to try to assess the effect of the delay in seeking treatment without reference to the histological type of growth involved. For example, delay is of little importance for patients with highly malignant (Grade III) tumours ; it does not seem to matter whether such cases attend for treatment early (less than 6 weeks) or late (6 to 12 months), the prognosis is equally bad. On the other hand, the time factor appears to influence the outlook of women with growths whose behaviour is essentially more benign (Grade I). In this type of case a loss of more than 6 months means a substantial fall in the survival rate.

4. The site of the tumour in the breast was, generally speaking, found to exert no striking effect on prognosis. However, it is possible that the outlook for patients with growths of low grade malignancy may be influenced slightly by this feature, depending on whether the medial or lateral hemispheres are involved.

5. When the patients were considered as a whole the size of the primary growth was found to influence prognosis, the larger the tumour the lower the survival rate. It is in the intermediate grade of tumours that these differences are especially found, and it is cases of this grade that are largely responsible for the general result. Size was evidently of no prognostic importance for women with tumours of a low and also a high grade of malignancy. It did not appear to matter whether the growths were small or large, the outlook was uniformly good in the former and bad in the latter group. In contrast to this, the prognosis for neoplasms of an intermediate degree of malignancy showed marked deterioration with increase of size.

6. Brief reference has been made to the progress of cases of breast cancer when associated with pregnancy or lactation.

Note by T.E. Cowan, Esq., F.C.I.S., F.R.S.S.: The inferences drawn from the tables shown are statistically sound.

I must again express my indebtedness to Professor R. W. Scarff for introducing me to his system of histological grading of breast cancer and for encouragement ; to Dr. A. C. Thackray for kind advice ; to Mr. T. E. Cowan for checking the statistics, and to Miss J. Chambers, of the Follow-up department, for tracing the patients.

For the cases employed in this work I am grateful to the surgeons of the Middlesex Hospital and its War-time Sector Units, and to Professor B. W. Windeyer of the Meyerstein Institute of Radiotherapy.

The expenses of this investigation were defrayed by the British Empire Cancer Campaign.

### REFERENCES.

BARTLETT, E. I.—(1933) *West. J. Surg.*, **41**, 243.

BECKTON, H.—(1909) *Arch. Middx. Hosp. clin. Ser.*, **15**, 74.

BLOOM, H. J. G.—(1950) *Brit. J. Cancer*, **4**, 259.

BUSK, T., AND CLEMMESEN, J.—(1947) *Ibid.*, **1**, 345.

CADE, STANFORD.—(1950) 'Malignant Disease and Its Treatment by Radium,' Bristol (Wright), 2nd ed., **3**, pp. 75, 78.

CAMPICHE, P., AND LAZARUS-BARLOW, W. S.—(1905) *Arch. Middx. Hosp. clin. Ser.*, **5**, 83.

CHASE, H. C.—(1947) *Surg. Gynec. Obstet.*, **85**, 712.

DALAND, E. M.—(1927) *Ibid.*, **44**, 264.

DAVIS, H. H.—(1938) *Ann. Surg.*, **107**, 207.

DE CHOLNOKY, T.—(1943) *Surg. Gynec. Obstet.*, **77**, 55.

DIETRICH, G.—(1892) *Dtsch. Z. Chir.*, **33**, 471.

EGGERS, C., DE CHOLNOKY, T., AND JESSUP, D. S. D.—(1941) *Ann. Surg.*, **113**, 321.

EVANS, W. A., AND LEUCUTIA, T.—(1930) *Amer. J. Roentgenol.*, **24**, 673.

GESCHICKTER, C. F.—(1945) 'Diseases of the Breast,' 2nd ed. Philadelphia (Lippincott), p. 403.

GREENOUGH, R. B.—(1925) *J. Cancer Res.*, **9**, 453.

GREENWOOD, MAJOR—(1926) Rep. publ. Hlth. med. Subj., Lond., No. 33.
HAAGENSEN, C. D., AND STOUT, A. P.—(1943) *Ann. Surg.*, **118**, 859.
HANDLEY, W. S.—(1922) 'Cancer of the Breast,' 2nd ed. London (Murray), pp. 156, 254.—(1927) *Surg. Gynec. Obstet.*, **45**, 721.
HARNETT, W. L.—(1948) *Brit. J. Cancer*, **2**, 212.
HARRINGTON, S. W.—(1937) *Ann. Surg.*, **106**, 690.
*Idem*—(1946) *Surgery*, **19**, 154.
HAWKINS, J. W.—(1944) *J. nat. Cancer Inst.*, **4**, 445.
HOOPES, B. F., AND MCGRAW, A. B.—(1942) *Surgery*, **12**, 892.
KAAE, S.—(1948) *Acta radiol., Stockh.*, **29**, 475.
KUNATH, C. A.—(1940) *Arch. Surg.*, **41**, 66.
LANE-CLAYPON, J. E.—(1924) Rep. publ. Hlth. med. Subj., Lond., No. 28.—(1926) *Ibid.*, No. 32.—(1928) *Ibid.*, No. 51.
LAZARUS-BARLOW, W. S., AND LEEMING, J. H.—(1924) *Lancet*, ii, 266.
LEE, B. J.—(1931) *Arch. Surg.*, **23**, 85.
*Idem* AND STUBENBORD, J. G.—(1928) *Surg. Gynec. Obstet.*, **47**, 812.
LEES, J. G., AND PARK, W. W.—(1949) *Brit. J. Cancer*, **3**, 186.
LEWIS, D., AND REINHOFF, W. F.—(1932) *Ann. Surg.*, **95**, 336.
LUFF, A. P.—(1932) *Brit. med. J.*, i, 897.
MACCARTY, W. C., AND SISTRUNK, W. E.—(1922) *Ann. Surg.*, **75**, 61.
MACDONALD, I.—(1942) *Surg. Gynec. Obstet.*, **74**, 75.
MATTHEWS, F. S.—(1933) *Ann. Surg.*, **98**, 635.
NATHANSON, I. T., AND WELCH, C. E.—(1936) *Amer. J. Cancer*, **28**, 40.
PACK, G. T., AND LIVINGSTONE, E. M.—(1940) 'Treatment of Cancer and Allied Diseases.' New York (Hoeber), vol. 1, p. 699.
PATEY, D. H., AND SCARFF, R. W.—(1928) *Lancet*, i, 801.
PERRY, A. C.—(1925) *Brit. J. Surg.*, **13**, 39.
PUTZKI, P. S., AND SCULLY, J. H.—(1946) *Surg. Gynec. Obstet.*, **83**, 751.
RICHARDS, G. E.—(1948) *Brit. J. Radiol.*, **21**, 109.
SAPHIR, O., AND AMROMIN, G. D.—(1948) *Cancer*, **1**, 2.
SCARFF, R. W., AND HANDLEY, R. S.—(1938) *Lancet*, ii, 582.
STOUT, A. P.—(1932) 'Human Cancer.' Philadelphia (Lea and Febiger), p. 301.
TAYLOR, G. W.—(1936) *New Engl. J. Med.*, **215**, 1276.
TRUSCOTT, B. McN.—(1947) *Brit. J. Cancer*, **1**, 129.
WEVILL, L. B.—(1932) *Edinb. med. J.*, **39**, 714.
WYARD, S.—(1925) *Lancet*, i, 1179.